# Supplementary material for: Tomato Xylem Sap Hydrophobins Vdh4 and Vdh5 Are Important for Late Stages of Verticillium dahliae Plant Infection
Source: J Fungi (Basel). 2022 Nov 27;8(12):1252. doi: 10.3390/jof8121252 (PMC9783231; doi:10.3390/jof8121252)
Supplement: Supplementary file 1 [file jof-08-01252-s001.zip › jof-2011945_ImagesS1_221121.pdf]

Supplementary Material

Supplementary Images S1

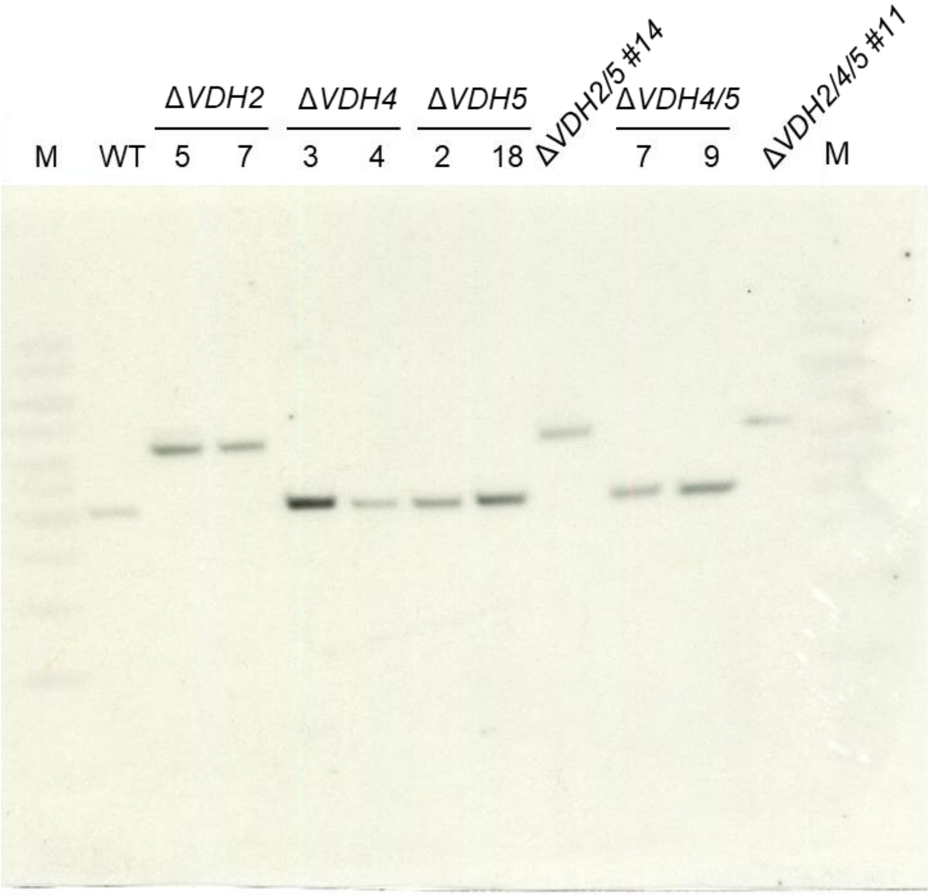

Image Figure S7a: Southern hybridization of *V. dahliae* wild-type (WT), as well as *VDH2*, *VDH4*, and *VDH5* single, double, and triple deletion strains with 3‘ *VDH2* as a probe. M: GeneRuler 1kb DNA Ladder (Thermo Fisher Scientific).

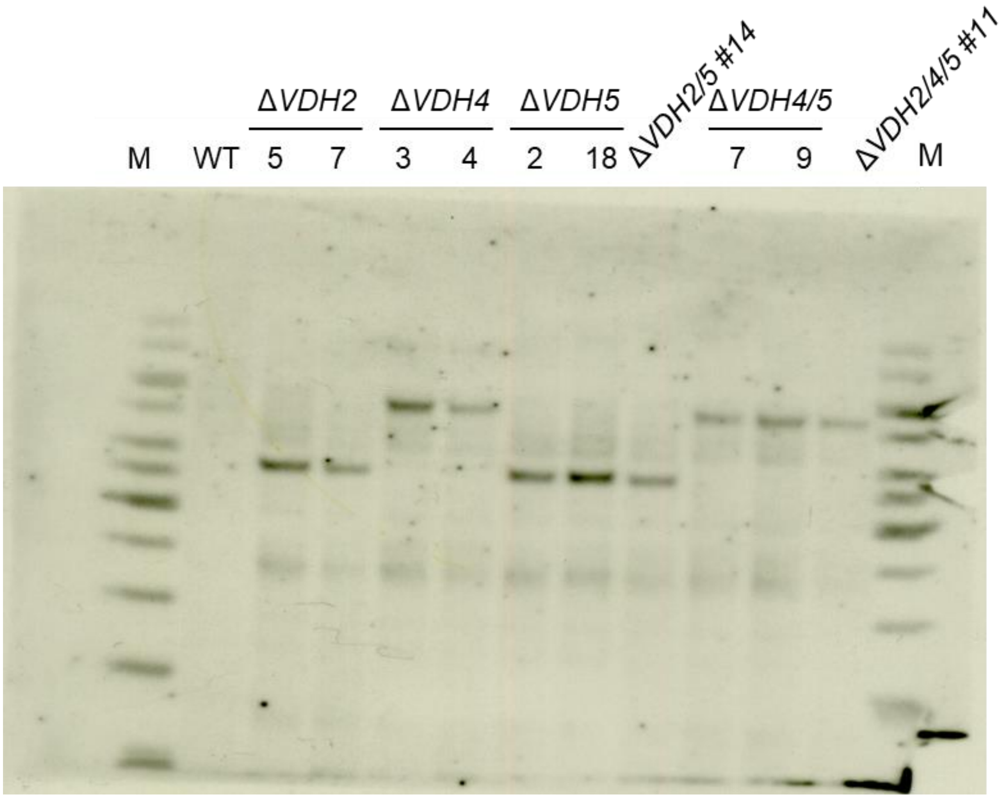

Image Figure S7b: Southern hybridization of *V. dahliae* wild-type (WT), as well as *VDH2*, *VDH4*, and *VDH5* single, double, and triple deletion strains with 3‘ *VDH4* as a probe. M: GeneRuler 1kb DNA Ladder (Thermo Fisher Scientific).

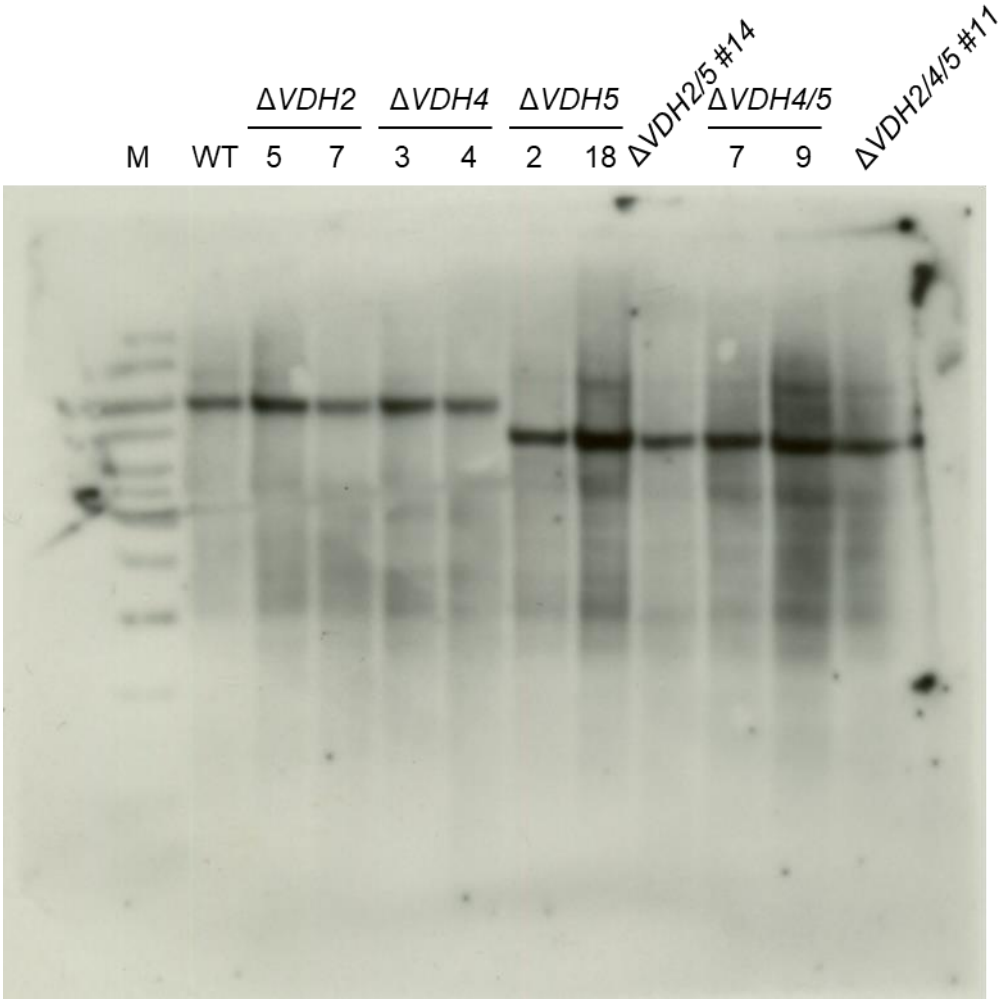

Image Figure S7c: Southern hybridization of *V. dahliae* wild-type (WT), as well as *VDH2*, *VDH4*, and *VDH5* single, double, and triple deletion strains with 3‘ *VDH5* as a probe. M: GeneRuler 1kb DNA Ladder (Thermo Fisher Scientific).

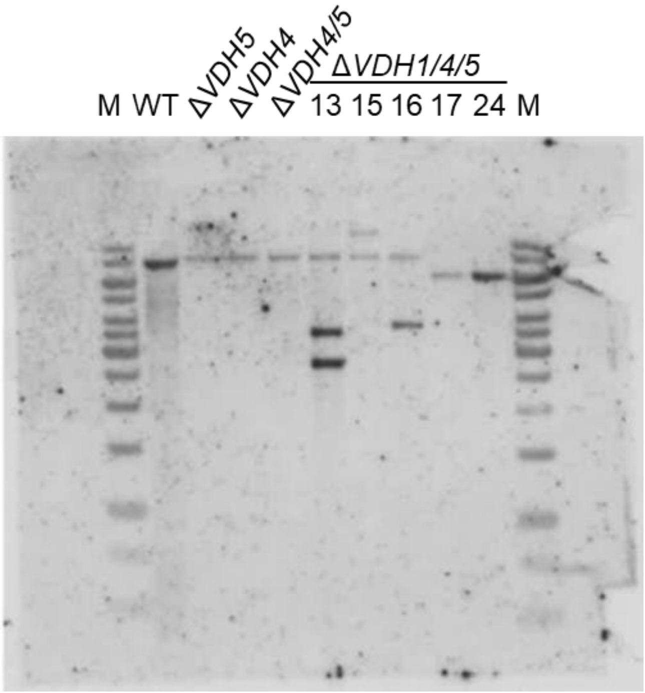

Image Figure S8a: Southern hybridization of *V. dahliae* wild-type (WT), *VDH4* and *VDH5* single and double deletion strains, as well as *VDH1/4/5* triple deletion strain with 5‘ *VDH1* as probe. M: GeneRuler 1kb DNA Ladder (Thermo Fisher Scientific).

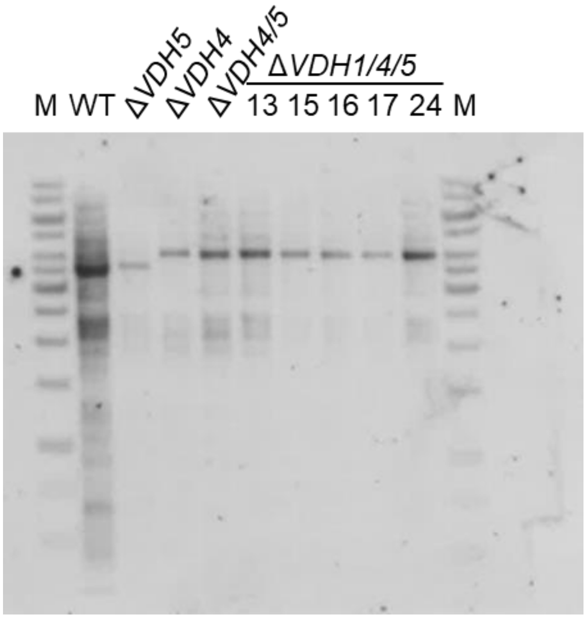

Image Figure S8b: Southern hybridization of *V. dahliae* wild-type (WT), *VDH4* and *VDH5* single and double deletion strains, as well as *VDH1/4/5* triple deletion strain with 3' *VDH4* as probe. M: GeneRuler 1kb DNA Ladder (Thermo Fisher Scientific).

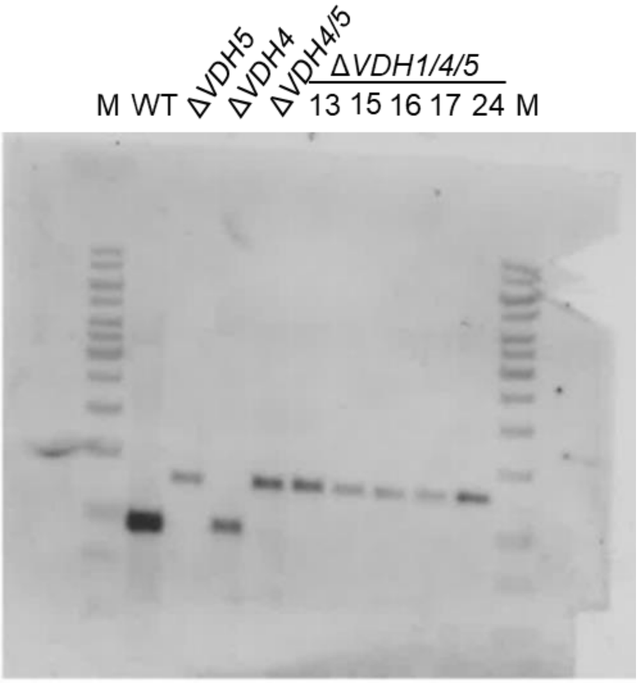

Image Figure S8c: Southern hybridization of *V. dahliae* wild-type (WT), *VDH4* and *VDH5* single and double deletion strains, as well as *VDH1/4/5* triple deletion strain with 3‘ *VDH5* as probe. M: GeneRuler 1kb DNA Ladder (Thermo Fisher Scientific).

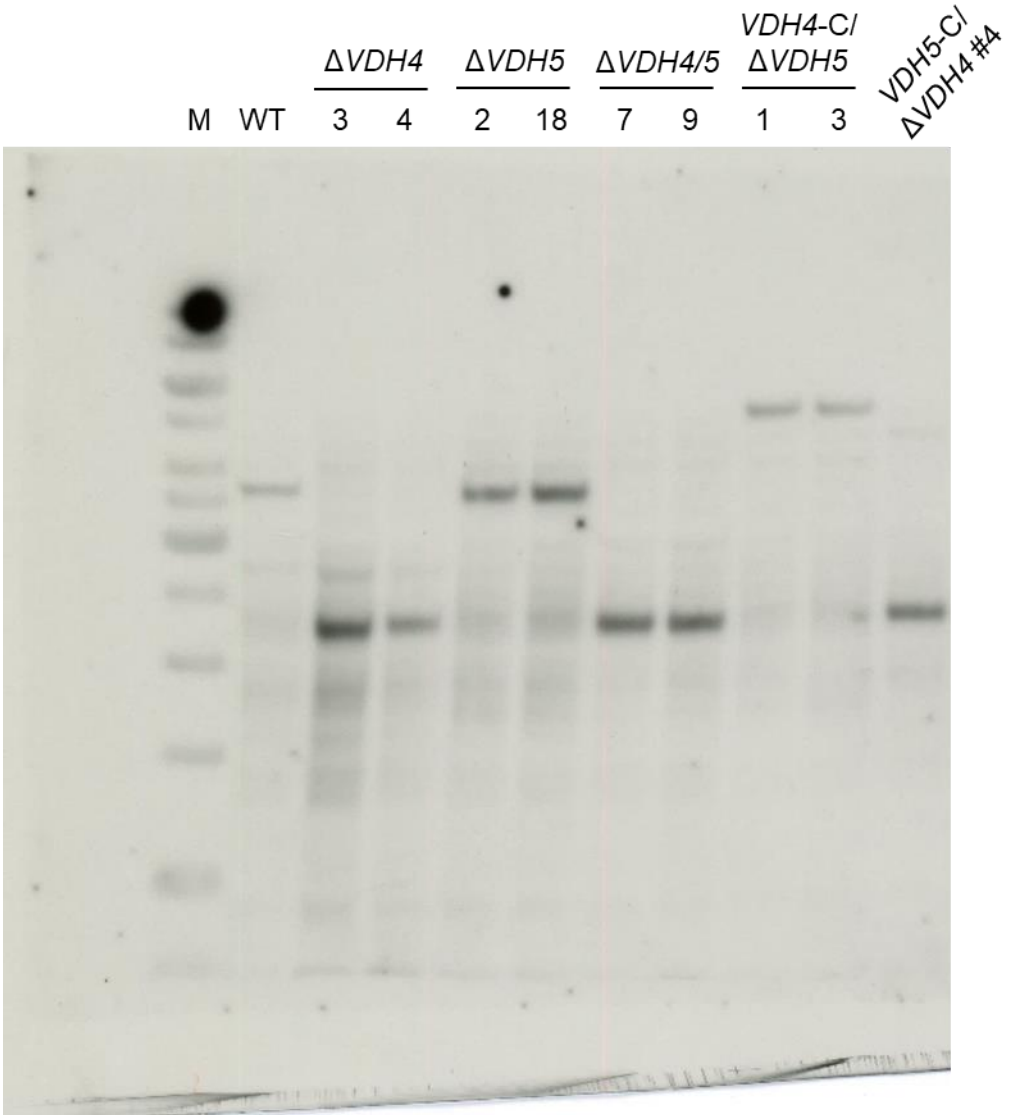

Image Figure S9a: Southern hybridization of *V. dahliae* wild-type (WT), as well as *VDH4* and *VDH5* deletion and complementation strains with 3‘ *VDH4* as a probe. M: GeneRuler 1kb DNA Ladder (Thermo Fisher Scientific).

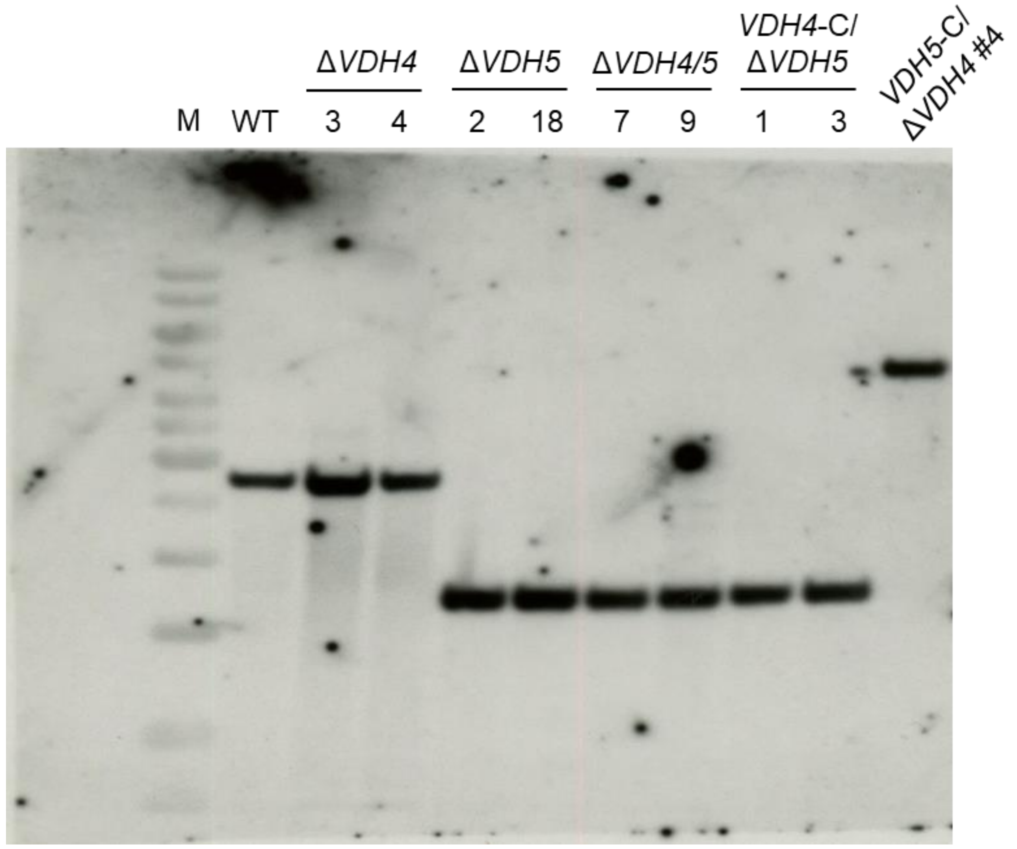

Image Figure S9b: Southern hybridization of *V. dahliae* wild-type (WT), as well as *VDH4* and *VDH5* deletion and complementation strains with 5‘ *VDH5* as a probe. M: GeneRuler 1kb DNA Ladder (Thermo Fisher Scientific).
