# Supplementary material for: Tomato Xylem Sap Hydrophobins Vdh4 and Vdh5 Are Important for Late Stages of Verticillium dahliae Plant Infection
Source: J Fungi (Basel). 2022 Nov 27;8(12):1252. doi: 10.3390/jof8121252 (PMC9783231; doi:10.3390/jof8121252)
Supplement: Supplementary file 1 [file jof-08-01252-s001.zip › jof-2011945_Supplementary material.pdf]

Supplementary Material

Supplementary Figures

Figure S1

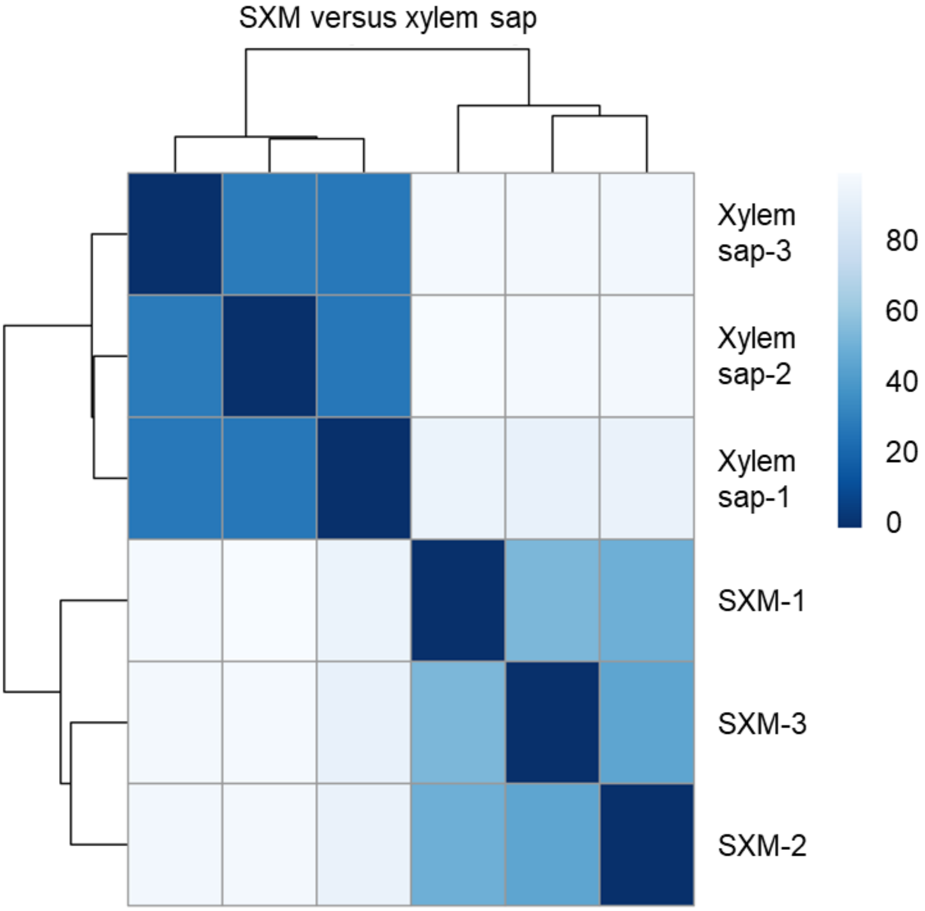

Figure S1: The similarity of *Verticillium dahliae* JR2 wild-type RNA samples grown in simulated xylem medium (SXM) or extracted tomato xylem sap is greater within than between groups. Liquid SXM was inoculated with 5×10^7 spores and incubated with shaking at 25 °C for five days. Mycelia were then transferred to either SXM or extracted tomato xylem sap and incubated for an additional eight hours. RNA was extracted from the mycelium in three biological replicates per condition and sequenced. Similarities within and between groups were determined based on the expression values of each sample. Shorter sample distances (indicated by darker blue) are measured for more closely related samples. Longer sample distances are represented by lighter blue. The three replicates under the same conditions were each clustered together, while they differed from the samples cultured under other conditions.

Figure S2

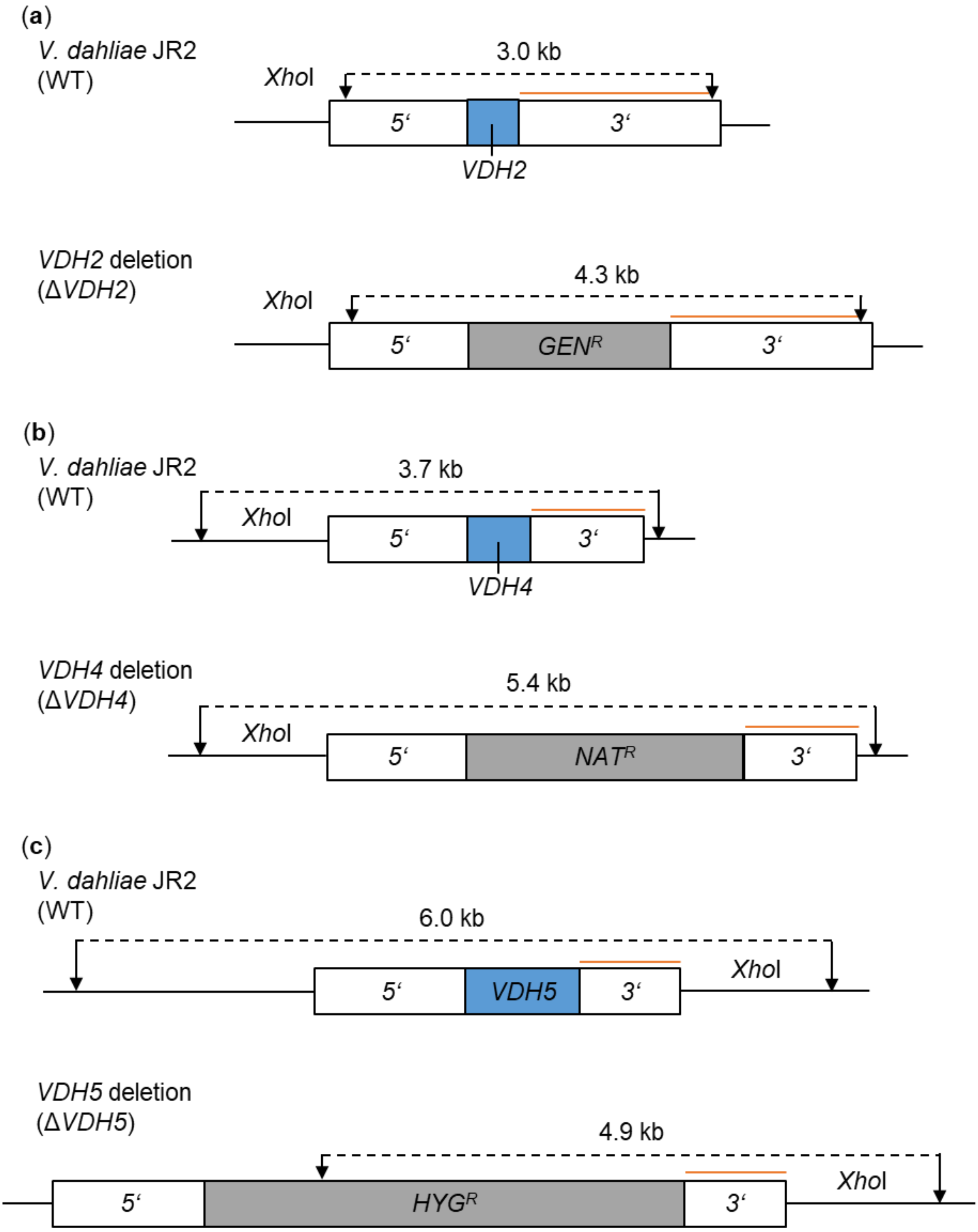

Figure S2: Scheme of restriction sites used for Southern hybridization of *V. dahliae* (a) *VDH2,* (b) *VDH4*, and (c) *VDH5* single, double and triple deletion strains. *V. dahliae* deletion strains of *VDH2* (Δ*VDH2*), *VDH4* (Δ*VDH4*), and *VDH5* (Δ*VDH5*), as well as the corresponding double (Δ*VDH2/5* and Δ*VDH4/5*) and triple (Δ*VDH2/4/5*) deletion strains were constructed by *Agrobacterium tumefaciens*-mediated transformation. The genomic region is replaced by homologous recombination of the up- and downstream flanking regions against a geneticin (*GEN*^{R}) resistance marker under control of a *trpC* promoter and terminator, a nourseothricin (*NAT*^{R}) or a hygromycin B (*HYG*^{R}) resistance marker under control of a *gpdA*

promoter and a *trpC* terminator. *Xho*I restriction sites are indicated by arrows. The 3’ flanking region was used as probe (highlighted in orange), and expected fragment lengths are indicated.

Figure S3

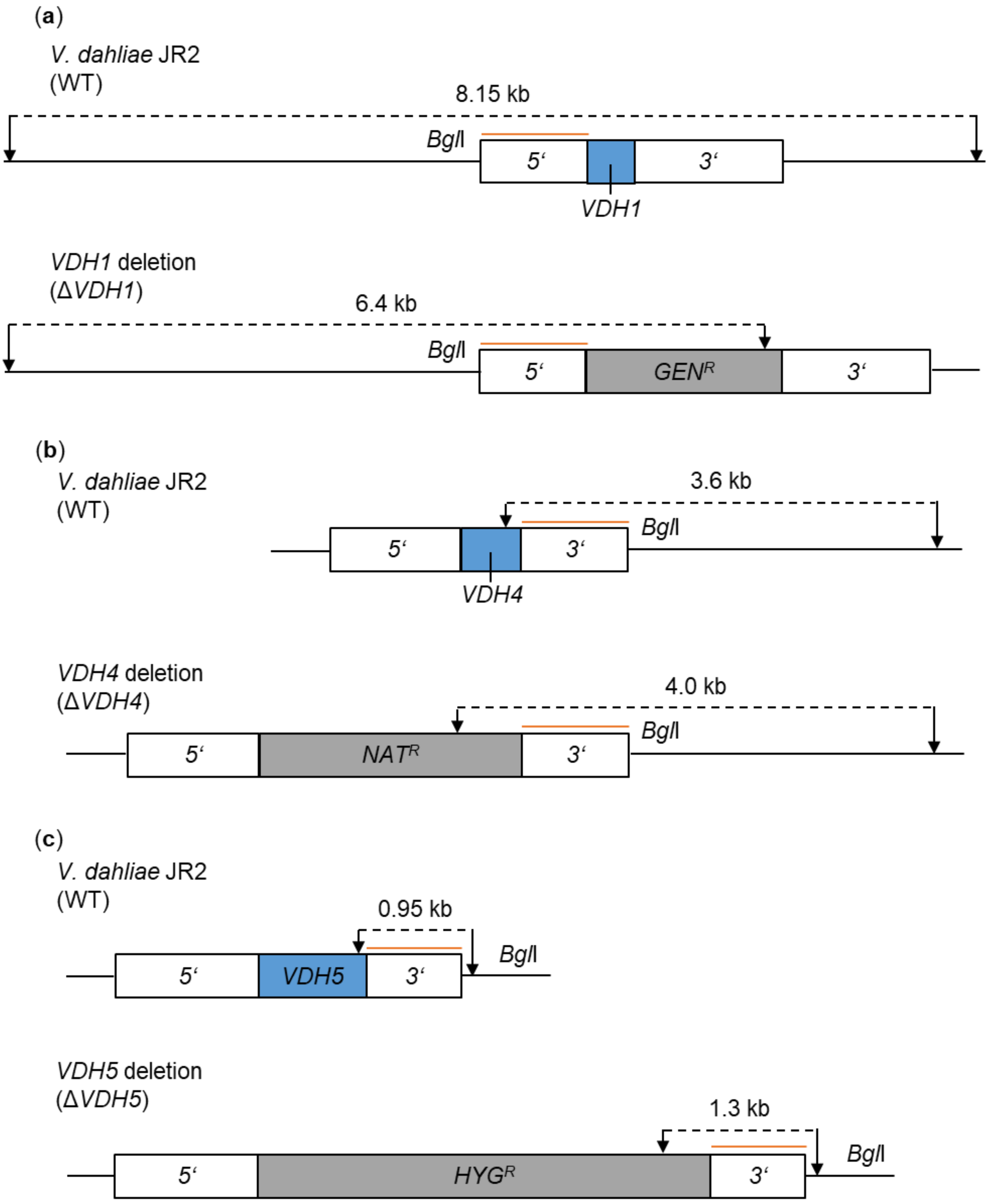

Figure S3: Scheme of restriction sites used for Southern hybridization of the *V. dahliae* *VDH1/4/5* triple deletion strain. The *V. dahliae* *VDH1* (Δ*VDH1*)/*VDH4* (Δ*VDH4*)/*VDH5* (Δ*VDH5*) triple deletion strain was constructed by *A. tumefaciens*-mediated transformation. The genomic region of each gene is replaced with a resistance marker cassette by homologous recombination of the up- and downstream flanking regions: geneticin (*GEN*^{R}) for *VDH1* (a), nourseothricin (*NAT*^{R}) for *VDH4* (b), and hygromycin B (*HYG*^{R}) for *VDH5* (c). Restriction sites of *Bgl*I are indicated by arrows, and the expected fragment lengths labeled with the 5’ flanking

region of *VDH1* or the 3’ flanking region of *VDH4* or *VDH5* as probes (indicated in orange) are given.

Figure S4

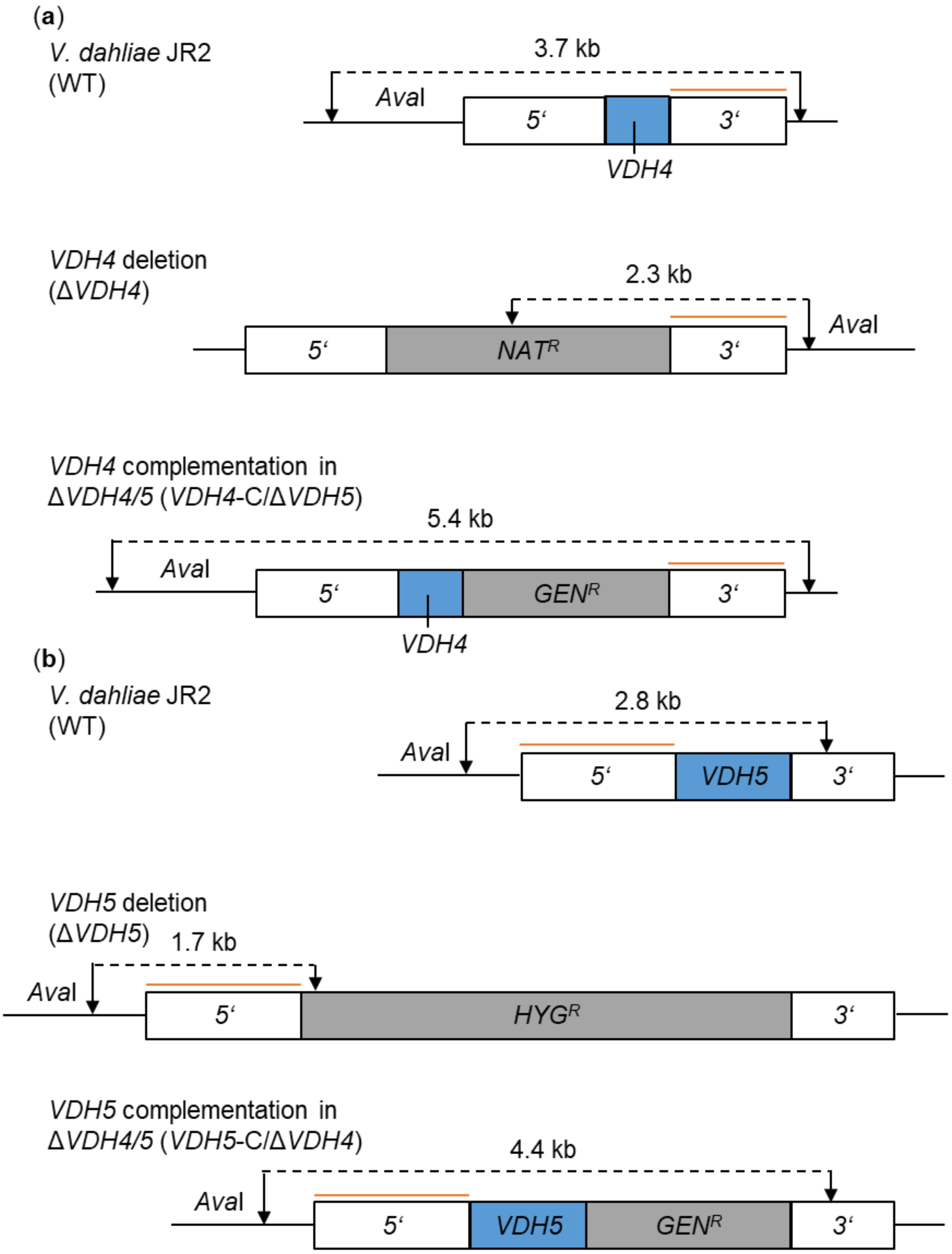

Figure S4: Scheme of restriction sites used for Southern hybridization of *V. dahliae* (a) *VDH4* and (b) *VDH5* deletion and complementation strains. The *V. dahliae VDH4* (Δ*VDH4*) and *VDH5* (Δ*VDH5*) deletion, and corresponding *VDH4* (*VDH4*-C/Δ*VDH5*) and *VDH5* (*VDH5*-C/Δ*VDH4*) complementation strains, were produced by *A. tumefaciens*-mediated transformation. The genomic region is replaced by homologous recombination of the up- and downstream flanking regions against a nourseothricin (*NAT*^{R}) in the case of *VDH4* or a hygromycin B (*HYG*^{R}) resistance marker in case of *VDH5* under the control of a *gpdA* promoter and a *trpC* terminator. For complementations, *VDH4* or *VDH5* was reintroduced into the

VDH4/5 double deletion strain at the genomic locus along with a geneticin (*GEN*^{R}) resistance marker under the control of a *trpC* promoter and terminator. *Ava*I restriction sites are indicated by arrows. Given are the expected fragment lengths labeled with the 3' flanking region of *VDH4* or the 5' flanking region of *VDH5* as probes (highlighted in orange).

Figure S5

1	ATG	AAG	TTC	ACC	ACT	GTC	GCC	GTC	GCC	TTC	30
31	TTC	GTC	GGC	CTT	GCC	GCT	GCC	CTC	CCC	ACC	60
61	ACC	GAC	TCC	TAC	ACT	CCT	GCG	CCC	TAC	GGT	90
91	GGC	AAC	GGT	GGA	CAC	AAC	GGA	GGC	CAC	AAC	120
121	GGT	GGT	AAC	AAC	GGA	GGC	CAC	AAC	GGT	GGT	150
151	CAC	AAC	GGC	GGC	AAT	GGT	GGC	AAC	AAT	GGT	180
181	GGC	AAT	GGT	GGT	AAC	GGC	AAC	AAC	GGA	GGC	210
211	AAC	AAC	GGA	GGC	AAC	AAC	GGA	GGC	AAC	AAC	240
241	GGA	GGC	AAC	AAC	GGA	GGC	AAC	AAC	GGA	GGC	270
271	AAC	AAC	GGA	GGC	AAT	GGC	GGT	AAC	AAC	GGC	300
301	GGC	AAT	GGT	GGA	AAC	AAC	GGC	GGA	AAC	GGT	330
331	GGC	AAT	GGA	AAC	AAC	GGT	GGA	AAC	GGA	AAC	360
361	AAC	GGC	GGC	AAT	GGC	GGC	AAC	AAC	GGT	GGT	390
391	AAC	AAC	GGC	GGT	AAC	AAC	GGA	GGC	AAC	GGC	420
421	GGA	AAC	AAC	GGC	GGC	AAT	GGT	GGA	AAC	AAT	450
451	GGC	GGC	AAC	AAC	GGA	GGC	AAC	GGC	GGA	AAC	480
481	AAC	GGT	GGC	AAC	AAC	GGA	GGC	AAC	AAC	GGA	510
511	GGC	AAC	GGC	GGT	AAC	AAC	GAC	TAC	GCG	CCT	540
541	TGC	CCT	TCC	GGC	CTC	AGC	ATG	ACC	CCC	GTC	570
571	TGC	TGC	GCT	ACT	AAC	GTC	CTC	GGC	CTT	CTC	600
601	GCT	CTC	AAC	TGC	GAT	GCT	CCC	AGC	AAG	ACT	630
631	CCC	ACC	TCG	GCC	AAG	GAC	TTC	CAG	AAG	ATC	660
661	TGC	GCC	GAC	GCC	GGC	ACG	GCC	GCC	AAG	TGC	690
691	TGC	ACC	CTC	AAC	CTC	CTC	AAC	CAG	GGT	GTC	720
721	CTG	TGC	CAG	GTT	CCC	GTT	GGC	GTC	GCC	GCC	750
751	TAA										753

Figure S5: The cDNA sequence of *V. dahliae* JR2 *VDH5*. The *VDH5* cDNA sequence of 753 bp was obtained by sequencing. For this purpose, RNA was extracted from mycelium of JR2 wild-type grown in liquid simulated xylem medium at 25 °C for five days and then in extracted tomato xylem sap for eight hours. The cDNA was synthesized using the QuantiTect Reverse Transcription Kit (Qiagen). *VDH5* was amplified with primers IM52 and IM53, and fully sequenced.

Figure S6

1	MKFTTVAVAF	FVGLAAALPT	TDSYTPAPYG	GNGGHNGGHN	40
41	GGNNGGHNGG	HNGGNGGNNG	GNGGNGNNGG	NNGGNNGGNN	80
81	GGNNGGNNGG	NNGGNGGNNG	GNGGNNGGNG	GNGNNGGNGN	120
121	NGGNGGNNGG	NNGGNNGGNG	GNNGGNGGNN	GGNNGGNGGN	160
161	NGGNNGGNNG	GNGGNNDYAP	CPSGLSMTPV	CCATNVLGLL	200
201	ALNCDAPSKT	PTSAKDFQKI	CADAGTAAKC	CTLNLLNQGV	240
241	LCQVPVGVAA.				250

Figure S6: Amino acid sequence of *V. dahliae* JR2 Vdh5. The sequence was derived from the corrected *VDH5* cDNA sequence obtained by sequencing (Figure S4). The Vdh5 protein consists of 250 amino acids (aa) and contains a predicted N-terminal signal peptide (1-17 aa, in orange), a dense granule Gra6 protein domain (19-142 aa, in gray) and a C-terminal domain of the cerato-ulmin hydrophobin family (180-245 aa, in blue).

Figure S7

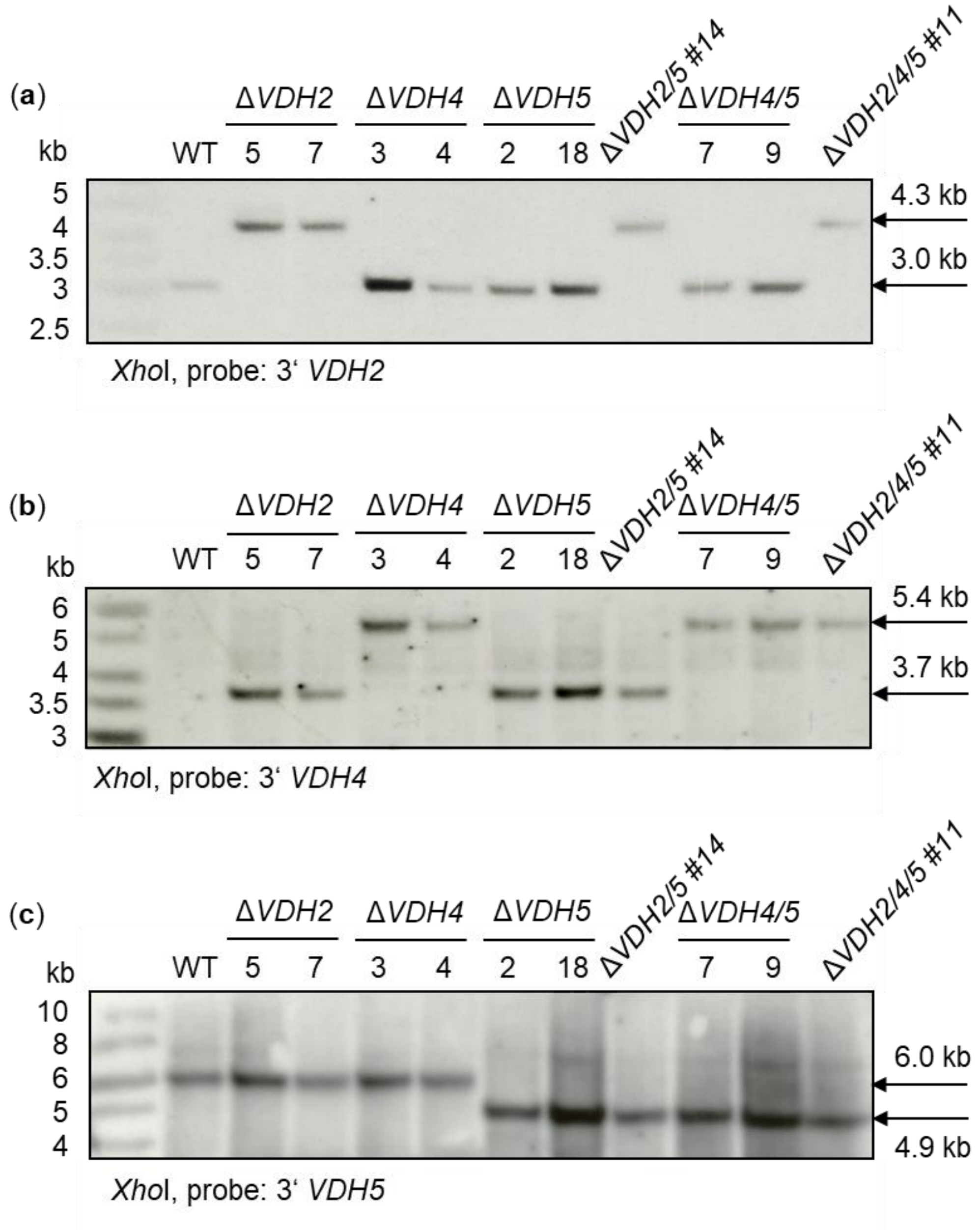

Figure S7: Verification of *V. dahliae VDH2, VDH4*, and *VDH5* single, double and triple deletion strains by Southern hybridization. The following strains were constructed and confirmed: Δ*VDH2* transformants numbers 5 (VGB648) and 7 (VGB649), Δ*VDH4* transformants numbers 3 (VGB566) and 4 (VGB567), Δ*VDH5* transformants numbers 2 (VGB568) and 18 (VGB569), Δ*VDH2/5* transformant number 14 (VGB651), Δ*VDH4/5* transformants numbers 7 (VGB623) and 9 (VGB624), and Δ*VDH2/4/5* transformant number 11 (VGB657). JR2 wild-type (WT) genomic DNA served as a control. The 3' flanking regions of (a) *VDH2*, (b) *VDH4*, or (c) *VDH5* were used as probes. The restriction enzyme *Xho*I was used to cut the genomic DNA. Signals corresponding to the predicted DNA fragment lengths (see also scheme in Figure S2) were observed in both wild-type and all deletion strains.

Figure S8

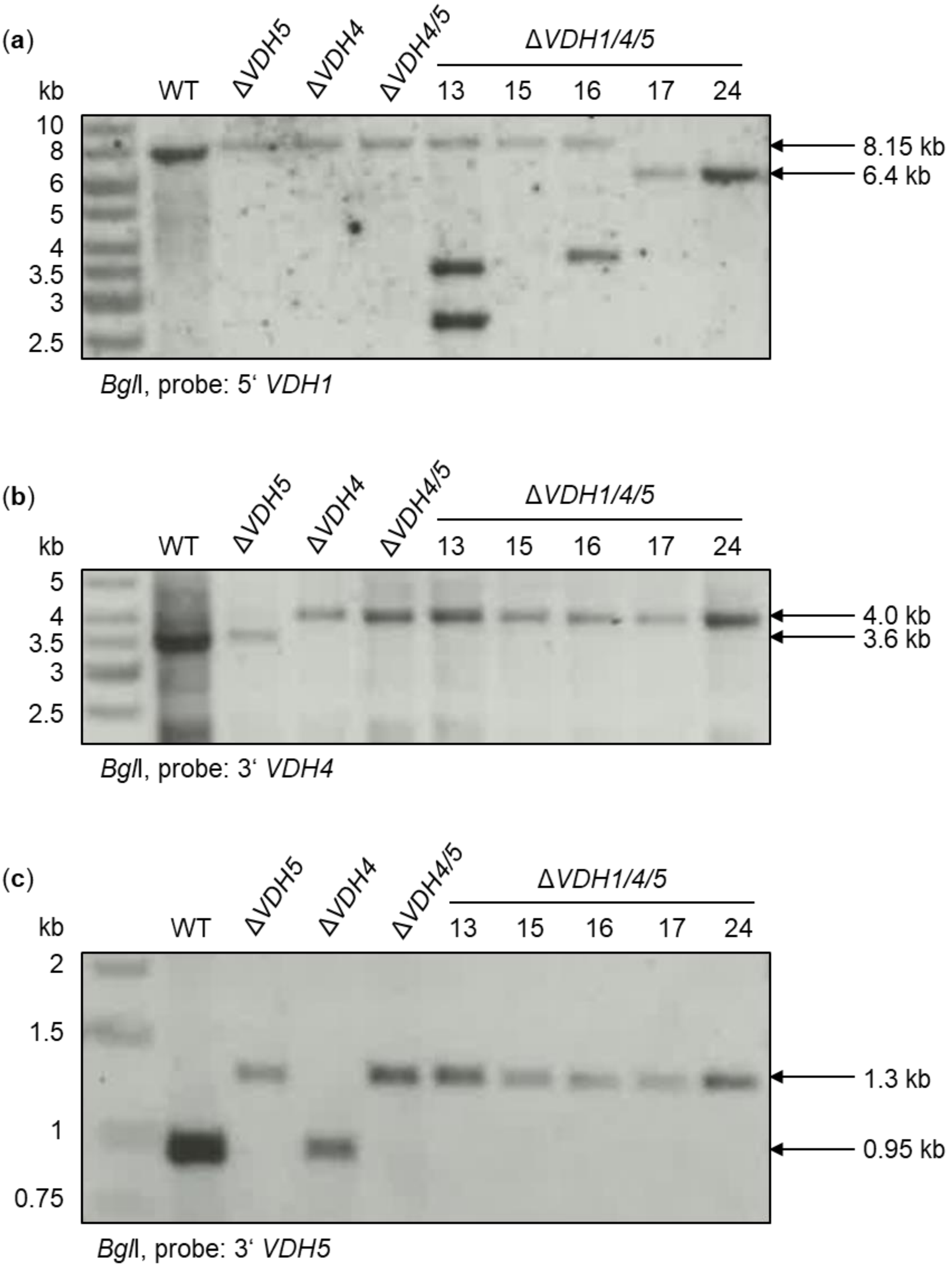

Figure S8: Verification of the *V. dahliae VDH1/4/5* triple deletion strain. Southern hybridization was performed to confirm the constructed Δ*VDH1/4/5* triple deletion transformants numbers 17 (VGB646) and 24 (VGB647). Genomic DNA from *V. dahliae* JR2 wild-type (WT) and single *VDH4* (Δ*VDH4*) and *VDH5* (Δ*VDH*) deletion strains, as well as the *VDH4/5* double deletion strain (Δ*VDH4*/5), served as controls. The 5' flanking region of (a) *VDH1* and the 3' flanking region of (b) *VDH4* and (c) *VDH5,* respectively, were used as probes, and genomic DNA was cut using the restriction enzyme *Bgl*I. Predicted signals corresponding

to DNA fragment length (see also scheme in Figure S3) were observed for both wild-type and all deletion strains.

Figure S9

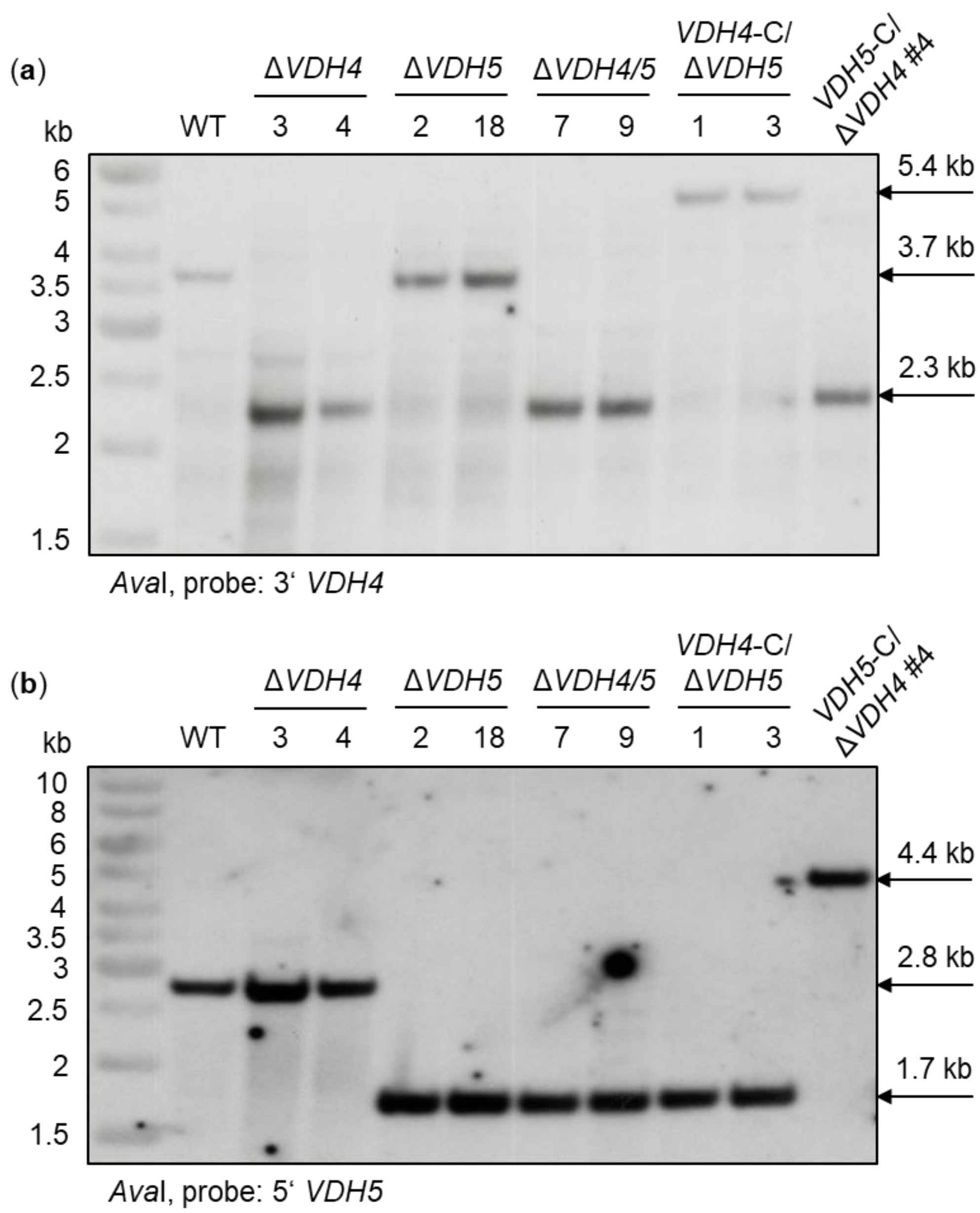

Figure S9: Verification of *V. dahliae VDH4* and *VDH5* deletion and complementation strains by Southern hybridization. The following strains were constructed and confirmed: Δ*VDH4* transformants numbers 3 (VGB566) and 4 (VGB567), Δ*VDH5* transformants numbers 2 (VGB568) and 18 (VGB569), Δ*VDH4/5* transformants numbers 7 (VGB623) and 9 (VGB624), *VDH4*-C/Δ*VDH5* (in VGB623) transformants numbers 1 (VGB690) and 3 (VGB691), and *VDH5*-C/Δ*VDH4* (in VGB623) transformant number 4 (VGB692). JR2 wild-type (WT) genomic DNA served as a control. The 3' flanking regions of (a) *VDH4* or the 5' flanking region of (b) *VDH5* were used as probes. The restriction enzyme *Ava*I was used to cut the genomic DNA. Signals corresponding to the predicted DNA fragment lengths (see also scheme in Figure S4) were observed for the wild-type and all deletion and complementation strains.

Figure S10

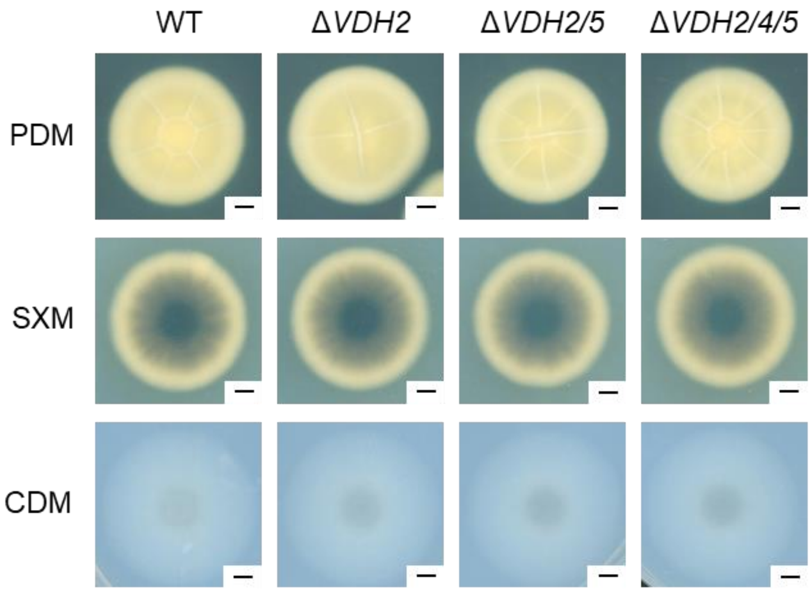

Figure S10: The presence of the hydrophobin-encoding genes *VDH2*, *VDH4*, and *VDH5* is dispensable for growth and microsclerotia production of *V. dahliae*. Potato dextrose medium (PDM), simulated xylem medium (SXM), and Czapek-Dox medium (CDM) plates were point-inoculated with 50,000 spores of the respective strains and incubated at 25 °C for ten days. Two independent experiments were conducted. Shown are scans from the bottom (scale = 0.5 cm). Vegetative growth and resting structure formation of the *VDH2* single deletion strain (Δ*VDH2*), as well as the *VDH2/5* double deletion (Δ*VDH2/5*) and the *VDH2/4/5* triple deletion (Δ*VDH2/4/5*) strains were compared with the JR2 wild-type (WT) and no differences were observed.

Figure S11

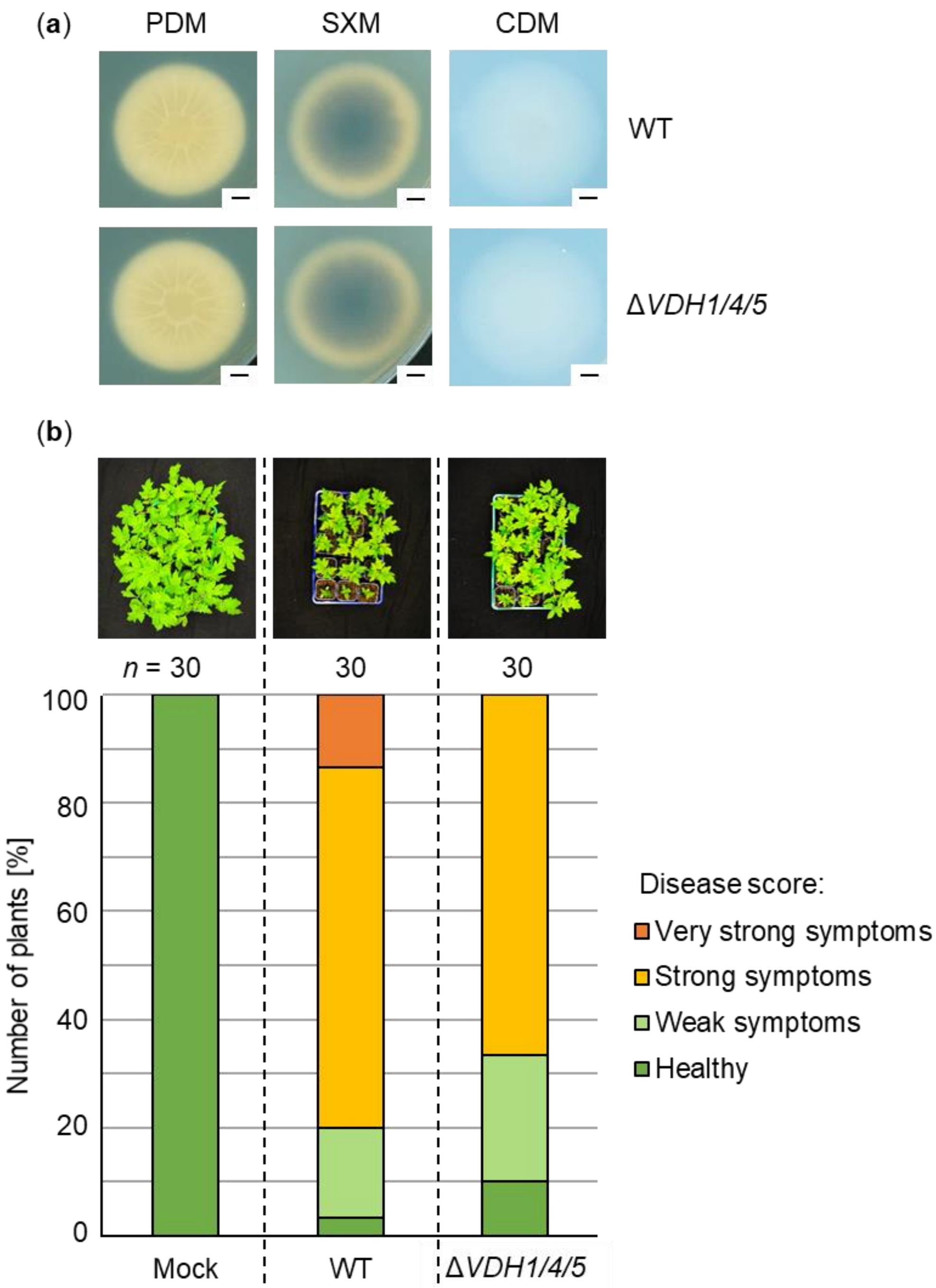

Figure S11: The presence of the hydrophobin-encoding gene *VDH1* is dispensable for the growth, microsclerotia production, and virulence of *V. dahliae*. *Ex-planta* and *in-planta* phenotypes of the *VDH1/4/5* triple deletion strain (Δ*VDH1/4/5*) were compared with the JR2 wild-type (WT). (a) Plates containing potato dextrose medium (PDM), simulated xylem medium (SXM), and Czapek-Dox medium (CDM), respectively, were point-inoculated with 50,000 spores of the indicated strains and incubated at 25 °C for ten days. Scans from the bottom (scale = 0.5 cm) reveal no differnces in vegetative growth and resting structure formation between the *VDH1/4/5* triple deletion strain and the wild-type. (b) Tomato seedlings (ten-day-old) were inoculated with spores of the wild-type or the *VDH1/4/5* triple deletion strain via root-dipping. Water-inoculated plants (mock) served as controls. Plants were incubated in a climate chamber at 16 h : 8 h light : dark at 22-25 °C. A general disease score was determined

21 days after inoculation based on plant height, length of longest leaf, and plant weight. The number of plants treated is indicated (*n*) and an overview of plants is shown. Plants infected by the wild-type exhibited stunted growth. In contrast, plants infected by the *VDH1/4/5* triple deletion strain did not show very strong disease symptoms, and about 15% more plants were healthy or only weakly infected.

Figure S12

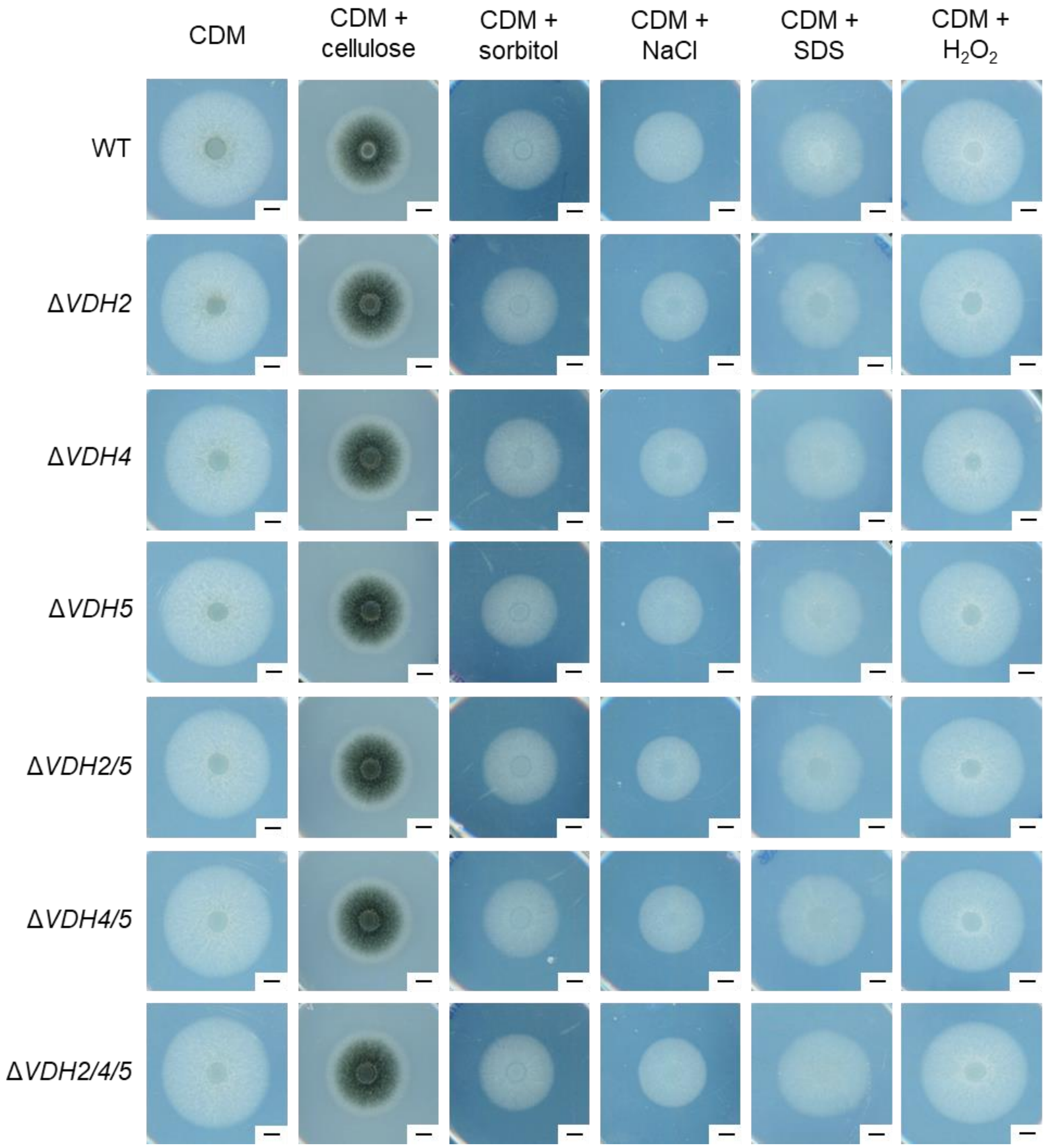

Figure S12: The absence of hydrophobins in *V. dahliae* leads to wild-type-like growth and microsclerotia formation under stress conditions. Vegetative growth and resting structure formation of *VDH2* (Δ*VDH2*), *VDH4* (Δ*VDH4*), and *VDH5* (Δ*VDH5*) single deletion strains, as well as *VDH2/5* (Δ*VDH2/5*) and *VDH4/5* (Δ*VDH4/5*) double deletion strains, and the *VDH2/4/5* triple deletion strain (Δ*VDH2/4/5*) were compared with JR2 wild-type (WT) on Czapek-Dox medium (CDM) supplemented with different stress-inducing agents. Indicated media plates were point-inoculated with 50,000 spores of the respective strains and incubated at 25 °C for ten days. Two independent experiments were performed. Scans from above depict a similar phenotype of Δ*VDH2*, Δ*VDH4,* Δ*VDH5*, Δ*VDH2/5*, Δ*VDH4/5*, and Δ*VDH2/4/5* compared with wild-type on all media tested (scale = 0.5 cm).

Figure S13

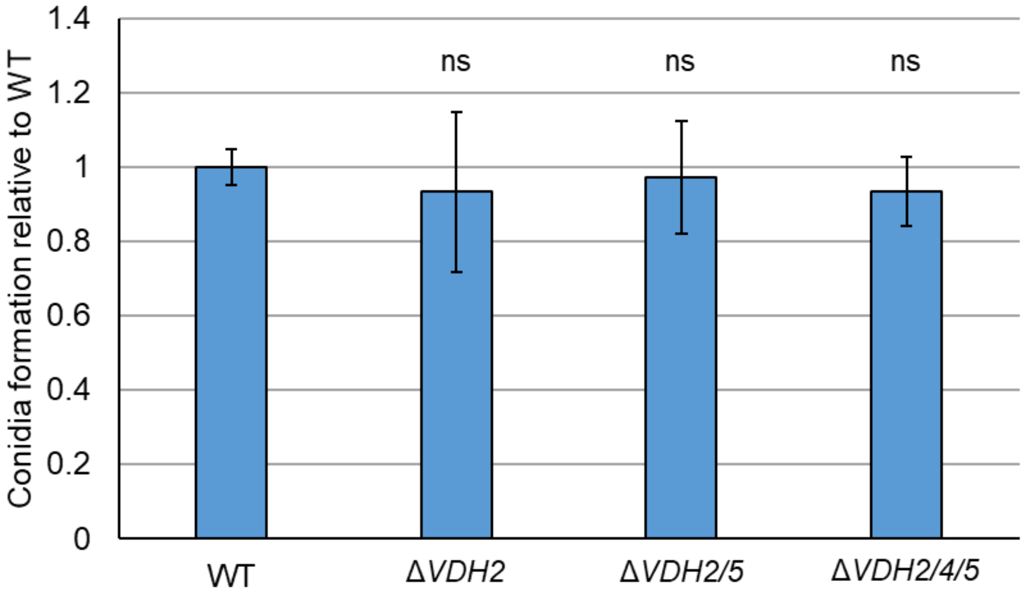

Figure S13: **Wild-type-like conidiospore levels in *V. dahliae* are independent of the presence of *VDH2*, *VDH4*, and *VDH5*.** Liquid simulated xylem medium was inoculated with 4,000 spores per ml and incubated at 25 °C under constant agitation for five days to quantify conidiation. Error bars represent standard deviations of the means, and significant differences from JR2 wild-type (WT) were calculated using Welch's *t*-tests. No difference in the ability to form conidiospores was detected between the *VDH2* single deletion (Δ*VDH2*), *VDH2/5* double deletion (Δ*VDH2/5*), *VDH2/4/5* triple deletion (Δ*VDH2/4/5*) strains, and the wild-type (ns, not significant). The experiment was performed three independent times, each with one or two biological (n = 4 to 6) and four technical replicates.

Supplementary Tables

Table S1: List of *V. dahliae* genes induced in tomato xylem sap found in significantly enriched categories by FunCat analysis.

Gene identifier	Protein name	FunCat description	log_2(fold change)
VDAG_JR2_Chr1g01070a	Putative uncharacterized protein	Allantoin and allantoate transport	4.75
VDAG_JR2_Chr1g01710a	Inner membrane transport protein yfaV	Allantoin and allantoate transport	1.52
VDAG_JR2_Chr1g18850a	Putative uncharacterized protein	Allantoin and allantoate transport	1.14
VDAG_JR2_Chr1g24150a	Uracil permease	Allantoin and allantoate transport	4.12
VDAG_JR2_Chr1g24720a	Allantoate permease	Allantoin and allantoate transport	1.03
VDAG_JR2_Chr1g24790a	High-affinity nicotinic acid transporter	Allantoin and allantoate transport	2.54
VDAG_JR2_Chr2g02540a	Pantothenate transporter liz1	Allantoin and allantoate transport	7.40
VDAG_JR2_Chr2g03860a	Inner membrane transport protein yfaV	Allantoin and allantoate transport	2.02
VDAG_JR2_Chr2g08730a	Putative uncharacterized protein	Allantoin and allantoate transport	1.43
VDAG_JR2_Chr2g13170a	Putative uncharacterized protein	Allantoin and allantoate transport	1.08
VDAG_JR2_Chr4g10900a	Vitamin H transporter	Allantoin and allantoate transport	3.04
VDAG_JR2_Chr4g12110a	High-affinity nicotinic acid transporter	Allantoin and allantoate transport	1.50
VDAG_JR2_Chr4g12390a	Putative uncharacterized protein	Allantoin and allantoate transport	8.31
VDAG_JR2_Chr6g02110a	Thiamine transporter	Allantoin and allantoate transport	1.23
VDAG_JR2_Chr6g04250a	Pantothenate transporter liz1	Allantoin and allantoate transport	3.63
VDAG_JR2_Chr6g09380a	Allantoate permease	Allantoin and allantoate transport	1.29
VDAG_JR2_Chr7g03220a	Allantoate permease	Allantoin and allantoate transport	1.78
VDAG_JR2_Chr8g01050a	Phthalate transporter	Allantoin and allantoate transport	1.36
VDAG_JR2_Chr8g02280a	Vitamin H transporter 1	Allantoin and allantoate transport	1.44
VDAG_JR2_Chr8g10350a	Phthalate transporter	Allantoin and allantoate transport	3.85
VDAG_JR2_Chr8g11110a	Allantoate permease	Allantoin and allantoate transport	1.96
VDAG_JR2_Chr1g05420a	Putative uncharacterized protein	Amino acid/amino acid derivatives transport	8.43
VDAG_JR2_Chr1g10700a	Solute carrier family 12 member 6	Amino acid/amino acid derivatives transport	2.26
VDAG_JR2_Chr1g11700a	Amino acid transporter	Amino acid/amino acid derivatives transport	1.11
VDAG_JR2_Chr1g12250a	Choline transport protein	Amino acid/amino acid derivatives transport	1.50

Gene identifier	Protein name	FunCat description	log_2(fold change)
VDAG_JR2_Chr1g25900a	Amino acid transporter	Amino acid/amino acid derivatives transport	1.42
VDAG_JR2_Chr2g02900a	High-affinity methionine permease	Amino acid/amino acid derivatives transport	1.66
VDAG_JR2_Chr2g04660a	Lysine-specific permease	Amino acid/amino acid derivatives transport	1.42
VDAG_JR2_Chr4g04640a	Aromatic and neutral aliphatic amino acid permease	Amino acid/amino acid derivatives transport	1.06
VDAG_JR2_Chr4g04720a	Proline-specific permease	Amino acid/amino acid derivatives transport	1.18
VDAG_JR2_Chr4g10760a	Choline transport protein	Amino acid/amino acid derivatives transport	4.55
VDAG_JR2_Chr4g11460a	Amino acid transporter	Amino acid/amino acid derivatives transport	1.47
VDAG_JR2_Chr4g11820a	General amino-acid permease GAP1	Amino acid/amino acid derivatives transport	2.99
VDAG_JR2_Chr4g12420a	Dicarboxylic amino acid permease	Amino acid/amino acid derivatives transport	2.70
VDAG_JR2_Chr5g06930a	Choline transport protein	Amino acid/amino acid derivatives transport	4.08
VDAG_JR2_Chr6g02110a	Thiamine transporter	Amino acid/amino acid derivatives transport	1.23
VDAG_JR2_Chr6g09120a	Aromatic and neutral aliphatic amino acid permease	Amino acid/amino acid derivatives transport	1.11
VDAG_JR2_Chr7g03180a	FMP42 protein	Amino acid/amino acid derivatives transport	4.22
VDAG_JR2_Chr7g07610a	Choline transport protein	Amino acid/amino acid derivatives transport	1.12
VDAG_JR2_Chr7g10310a	Amino-acid permease inda1	Amino acid/amino acid derivatives transport	1.13
VDAG_JR2_Chr8g01840a	Sugar transporter family protein	Amino acid/amino acid derivatives transport	1.33
VDAG_JR2_Chr8g09210a	Carboxylic acid transport protein	Amino acid/amino acid derivatives transport	7.57
VDAG_JR2_Chr1g00640a	Putative uncharacterized protein	Cation transport (H+, Na+, K+, Ca2+, NH4+, etc.)	1.08
VDAG_JR2_Chr1g02230a	Putative uncharacterized protein	Cation transport (H+, Na+, K+, Ca2+, NH4+, etc.)	1.27
VDAG_JR2_Chr1g02790a	Calcium-transporting ATPase	Cation transport (H+, Na+, K+, Ca2+, NH4+, etc.)	1.45
VDAG_JR2_Chr1g10700a	Solute carrier family 12 member 6	Cation transport (H+, Na+, K+, Ca2+, NH4+, etc.)	2.26
VDAG_JR2_Chr1g15800a	Cobalt uptake protein COT1	Cation transport (H+, Na+, K+, Ca2+, NH4+, etc.)	2.19
VDAG_JR2_Chr1g15850a	ABC transporter CDR4	Cation transport (H+, Na+, K+, Ca2+, NH4+, etc.)	1.22
VDAG_JR2_Chr1g18920a	Putative uncharacterized protein	Cation transport (H+, Na+, K+, Ca2+, NH4+, etc.)	1.06
VDAG_JR2_Chr1g22590a	Vacuolar calcium ion transporter	Cation transport (H+, Na+, K+, Ca2+, NH4+, etc.)	3.81
VDAG_JR2_Chr1g23670a	Putative uncharacterized protein	Cation transport (H+, Na+, K+, Ca2+, NH4+, etc.)	1.76
VDAG_JR2_Chr1g25420a	Urea active transporter	Cation transport (H+, Na+, K+, Ca2+, NH4+, etc.)	1.17

Gene identifier	Protein name	FunCat description	log_2(fold change)
VDAG_JR2_Chr1g26890a	Pleiotropic ABC multiple drug efflux transporter	Cation transport (H+, Na+, K+, Ca2+, NH4+, etc.)	1.07
VDAG_JR2_Chr1g27550a	Urea active transporter	Cation transport (H+, Na+, K+, Ca2+, NH4+, etc.)	2.12
VDAG_JR2_Chr1g29160a	Calcium-transporting ATPase	Cation transport (H+, Na+, K+, Ca2+, NH4+, etc.)	1.22
VDAG_JR2_Chr2g07940a	Phospholipid-transporting ATPase	Cation transport (H+, Na+, K+, Ca2+, NH4+, etc.)	1.15
VDAG_JR2_Chr2g12640a	Putative uncharacterized protein	Cation transport (H+, Na+, K+, Ca2+, NH4+, etc.)	1.14
VDAG_JR2_Chr2g13190a	Voltage-gated potassium channel subunit beta-3	Cation transport (H+, Na+, K+, Ca2+, NH4+, etc.)	1.20
VDAG_JR2_Chr3g05190a	Sodium/calcium exchanger protein	Cation transport (H+, Na+, K+, Ca2+, NH4+, etc.)	1.17
VDAG_JR2_Chr3g11010a	Vacuolar ATP synthase 98 kDa subunit	Cation transport (H+, Na+, K+, Ca2+, NH4+, etc.)	2.69
VDAG_JR2_Chr3g11520a	Potassium channel	Cation transport (H+, Na+, K+, Ca2+, NH4+, etc.)	1.06
VDAG_JR2_Chr4g04200a	Calcium permease	Cation transport (H+, Na+, K+, Ca2+, NH4+, etc.)	1.15
VDAG_JR2_Chr4g07840a	Potassium transporter 1	Cation transport (H+, Na+, K+, Ca2+, NH4+, etc.)	1.47
VDAG_JR2_Chr4g12160a	Ammonium transporter 1	Cation transport (H+, Na+, K+, Ca2+, NH4+, etc.)	2.22
VDAG_JR2_Chr5g01900a	High-affinity potassium transport protein	Cation transport (H+, Na+, K+, Ca2+, NH4+, etc.)	3.47
VDAG_JR2_Chr5g01910a	Na+/H+ antiporter	Cation transport (H+, Na+, K+, Ca2+, NH4+, etc.)	2.53
VDAG_JR2_Chr5g05790a	Membrane transporter	Cation transport (H+, Na+, K+, Ca2+, NH4+, etc.)	1.24
VDAG_JR2_Chr5g10400a	Calcium-channel protein cch1	Cation transport (H+, Na+, K+, Ca2+, NH4+, etc.)	1.30
VDAG_JR2_Chr5g12230a	Na+/H+ antiporter	Cation transport (H+, Na+, K+, Ca2+, NH4+, etc.)	1.49
VDAG_JR2_Chr6g01260a	Pantothenate transporter liz1	Cation transport (H+, Na+, K+, Ca2+, NH4+, etc.)	2.12
VDAG_JR2_Chr6g03600a	Low-affinity potassium transport protein	Cation transport (H+, Na+, K+, Ca2+, NH4+, etc.)	1.13
VDAG_JR2_Chr6g06420a	Multidrug resistance protein CDR2	Cation transport (H+, Na+, K+, Ca2+, NH4+, etc.)	1.11
VDAG_JR2_Chr6g07230a	Myb-like DNA-binding protein	Cation transport (H+, Na+, K+, Ca2+, NH4+, etc.)	1.71
VDAG_JR2_Chr6g09800a	K+/H+ antiporter 1	Cation transport (H+, Na+, K+, Ca2+, NH4+, etc.)	2.20
VDAG_JR2_Chr7g02750a	Low-affinity potassium transport protein	Cation transport (H+, Na+, K+, Ca2+, NH4+, etc.)	1.50
VDAG_JR2_Chr7g10440a	Sodium/nucleoside cotransporter	Cation transport (H+, Na+, K+, Ca2+, NH4+, etc.)	1.21
VDAG_JR2_Chr8g06660a	Ammonium transporter MEP1	Cation transport (H+, Na+, K+, Ca2+, NH4+, etc.)	1.29
VDAG_JR2_Chr8g09670a	ATP synthase subunit alpha	Cation transport (H+, Na+, K+, Ca2+, NH4+, etc.)	2.64
VDAG_JR2_Chr1g01080a	Maltose permease	C-compound and carbohydrate metabolism	2.77

Gene identifier	Protein name	FunCat description	log_2(fold change)
VDAG_JR2_Chr1g02220a	Sugar transporter	C-compound and carbohydrate metabolism	2.06
VDAG_JR2_Chr1g04070a	Estradiol 17-beta-dehydrogenase	C-compound and carbohydrate metabolism	1.49
VDAG_JR2_Chr1g05040a	Putative uncharacterized protein	C-compound and carbohydrate metabolism	1.48
VDAG_JR2_Chr1g05250a	NADP-dependent mannitol dehydrogenase	C-compound and carbohydrate metabolism	1.10
VDAG_JR2_Chr1g05370a	Rhamnolipids biosynthesis 3-oxoacyl-[acyl-carrier-protein] reductase	C-compound and carbohydrate metabolism	2.07
VDAG_JR2_Chr1g05390a	Aldehyde dehydrogenase	C-compound and carbohydrate metabolism	4.23
VDAG_JR2_Chr1g05500a	3-oxoacyl-[acyl-carrier-protein] reductase	C-compound and carbohydrate metabolism	1.17
VDAG_JR2_Chr1g05760a	Galactose-proton symporter	C-compound and carbohydrate metabolism	1.70
VDAG_JR2_Chr1g06300a	Oxidoreductase 2-nitropropane dioxygenase family	C-compound and carbohydrate metabolism	1.02
VDAG_JR2_Chr1g06670a	Alkaline phosphatase H	C-compound and carbohydrate metabolism	2.68
VDAG_JR2_Chr1g08876a	FAD binding domain-containing protein	C-compound and carbohydrate metabolism	1.12
VDAG_JR2_Chr1g13450a	4-coumarate-CoA ligase	C-compound and carbohydrate metabolism	1.26
VDAG_JR2_Chr1g15180a	Isocitrate lyase	C-compound and carbohydrate metabolism	1.33
VDAG_JR2_Chr1g15790a	Versicolorin reductase	C-compound and carbohydrate metabolism	1.03
VDAG_JR2_Chr1g15970a	Amine oxidase	C-compound and carbohydrate metabolism	1.07
VDAG_JR2_Chr1g17290a	Alpha-galactosidase (EC 3.2.1.22) (Melibiase)	C-compound and carbohydrate metabolism	1.10
VDAG_JR2_Chr1g18220a	Pisatin demethylase	C-compound and carbohydrate metabolism	1.58
VDAG_JR2_Chr1g18710a	Glucosamine 6-phosphate N-acetyltransferase	C-compound and carbohydrate metabolism	1.02
VDAG_JR2_Chr1g19160a	Tyrosyl-DNA phosphodiesterase	C-compound and carbohydrate metabolism	1.03
VDAG_JR2_Chr1g20150a	Oxidoreductase 2-nitropropane dioxygenase family	C-compound and carbohydrate metabolism	1.04
VDAG_JR2_Chr1g20550a	Neuroligin-4	C-compound and carbohydrate metabolism	1.75
VDAG_JR2_Chr1g21570a	Beta-galactosidase (EC 3.2.1.23)	C-compound and carbohydrate metabolism	1.68
VDAG_JR2_Chr1g21690a	Peroxisomal (S)-2-hydroxy-acid oxidase	C-compound and carbohydrate metabolism	4.12

Gene identifier	Protein name	FunCat description	log_2(fold change)
VDAG_JR2_Chr1g22650a	D-arabinono-1,4-lactone oxidase	C-compound and carbohydrate metabolism	1.84
VDAG_JR2_Chr1g23240a	Mixed-linked glucanase	C-compound and carbohydrate metabolism	1.88
VDAG_JR2_Chr1g23860a	Putative uncharacterized protein	C-compound and carbohydrate metabolism	2.55
VDAG_JR2_Chr1g24130a	Polygalacturonase	C-compound and carbohydrate metabolism	1.98
VDAG_JR2_Chr1g24570a	Pristinamycin IIA synthase subunit A	C-compound and carbohydrate metabolism	3.01
VDAG_JR2_Chr1g26170a	Putative uncharacterized protein	C-compound and carbohydrate metabolism	1.22
VDAG_JR2_Chr1g26510a	Cholinesterase	C-compound and carbohydrate metabolism	2.46
VDAG_JR2_Chr1g26770a	Sorbitol dehydrogenase	C-compound and carbohydrate metabolism	2.27
VDAG_JR2_Chr1g27050a	Ubiquinone/menaquinone biosynthesis methyltransferase ubiE	C-compound and carbohydrate metabolism	2.93
VDAG_JR2_Chr1g28200a	Putative uncharacterized protein	C-compound and carbohydrate metabolism	1.29
VDAG_JR2_Chr1g28650a	3-hydroxyisobutyrate dehydrogenase	C-compound and carbohydrate metabolism	1.13
VDAG_JR2_Chr1g28970a	Alpha-galactosidase (EC 3.2.1.22) (Melibiase)	C-compound and carbohydrate metabolism	2.20
VDAG_JR2_Chr2g00430a	Pectate lyase	C-compound and carbohydrate metabolism	2.89
VDAG_JR2_Chr2g01760a	Putative uncharacterized protein	C-compound and carbohydrate metabolism	1.44
VDAG_JR2_Chr2g03770a	6-phosphogluconate dehydrogenase, decarboxylating (EC 1.1.1.44)	C-compound and carbohydrate metabolism	1.23
VDAG_JR2_Chr2g04160a	4-coumarate-CoA ligase	C-compound and carbohydrate metabolism	2.58
VDAG_JR2_Chr2g05040a	3-alpha-(Or 20-beta)-hydroxysteroid dehydrogenase	C-compound and carbohydrate metabolism	1.25
VDAG_JR2_Chr2g05680a	Putative uncharacterized protein	C-compound and carbohydrate metabolism	1.17
VDAG_JR2_Chr2g05870a	Glucoamylase (EC 3.2.1.3) (1,4-alpha-D-glucan glucohydrolase) (Glucan 1,4-alpha-glucosidase)	C-compound and carbohydrate metabolism	1.49
VDAG_JR2_Chr2g06830a	Xylosidase/arabinosidase	C-compound and carbohydrate metabolism	1.04
VDAG_JR2_Chr2g08750a	FAD/FMN-containing dehydrogenase	C-compound and carbohydrate metabolism	2.33
VDAG_JR2_Chr2g08810a	Endoglucanase	C-compound and carbohydrate metabolism	1.60
VDAG_JR2_Chr2g08880a	Maltose permease MAL31	C-compound and carbohydrate metabolism	1.34

Gene identifier	Protein name	FunCat description	log_2(fold change)
VDAG_JR2_Chr2g11080a	3-alpha-(Or 20-beta)-hydroxysteroid dehydrogenase	C-compound and carbohydrate metabolism	1.34
VDAG_JR2_Chr2g12320a	General alpha-glucoside permease	C-compound and carbohydrate metabolism	1.43
VDAG_JR2_Chr2g12790a	FAD binding domain-containing protein	C-compound and carbohydrate metabolism	2.69
VDAG_JR2_Chr2g13240a	Choline dehydrogenase	C-compound and carbohydrate metabolism	4.55
VDAG_JR2_Chr2g13290a	Pectate lyase D	C-compound and carbohydrate metabolism	1.97
VDAG_JR2_Chr3g01050a	Membrane transporter D1	C-compound and carbohydrate metabolism	1.34
VDAG_JR2_Chr3g02150a	Chitinase	C-compound and carbohydrate metabolism	1.17
VDAG_JR2_Chr3g02970a	Mixed-linked glucanase	C-compound and carbohydrate metabolism	1.93
VDAG_JR2_Chr3g03180a	Oligo-1,6-glucosidase	C-compound and carbohydrate metabolism	1.19
VDAG_JR2_Chr3g03230a	4-coumarate-CoA ligase	C-compound and carbohydrate metabolism	1.49
VDAG_JR2_Chr3g03460a	Alpha-mannosidase	C-compound and carbohydrate metabolism	1.12
VDAG_JR2_Chr3g03540a	Putative uncharacterized protein	C-compound and carbohydrate metabolism	1.02
VDAG_JR2_Chr3g06580a	cAMP-regulated D2 protein	C-compound and carbohydrate metabolism	1.50
VDAG_JR2_Chr3g07650a	Acetate kinase	C-compound and carbohydrate metabolism	1.13
VDAG_JR2_Chr3g09290a	D-3-phosphoglycerate dehydrogenase	C-compound and carbohydrate metabolism	1.70
VDAG_JR2_Chr3g10320a	Beta-hexosaminidase	C-compound and carbohydrate metabolism	1.47
VDAG_JR2_Chr3g10330a	N-acetylglucosamine-6-phosphate deacetylase	C-compound and carbohydrate metabolism	1.43
VDAG_JR2_Chr3g10350a	Glucosamine-6-phosphate isomerase	C-compound and carbohydrate metabolism	2.27
VDAG_JR2_Chr3g10360a	Hexokinase-1	C-compound and carbohydrate metabolism	1.49
VDAG_JR2_Chr3g10580a	Glycosyl hydrolases family 32 superfamily	C-compound and carbohydrate metabolism	2.43
VDAG_JR2_Chr3g10590a	General alpha-glucoside permease	C-compound and carbohydrate metabolism	1.66
VDAG_JR2_Chr3g10840a	Short-chain dehydrogenase/reductase SDR	C-compound and carbohydrate metabolism	1.19
VDAG_JR2_Chr3g11080a	Endo-1,4-beta-xylanase	C-compound and carbohydrate metabolism	1.36
VDAG_JR2_Chr3g12210a	Lipase	C-compound and carbohydrate metabolism	4.43
VDAG_JR2_Chr3g12300a	Pectate lyase	C-compound and carbohydrate metabolism	2.07
VDAG_JR2_Chr3g12310a	Arabinose 5-phosphate isomerase	C-compound and carbohydrate metabolism	2.80

Gene identifier	Protein name	FunCat description	log_2(fold change)
VDAG_JR2_Chr3g12360a	Cellobiose dehydrogenase	C-compound and carbohydrate metabolism	2.27
VDAG_JR2_Chr3g12560a	Maltose permease MAL31	C-compound and carbohydrate metabolism	1.21
VDAG_JR2_Chr3g12810a	FAD binding domain-containing protein	C-compound and carbohydrate metabolism	1.55
VDAG_JR2_Chr3g12900a	Alpha-glucosides permease MPH2/3	C-compound and carbohydrate metabolism	1.08
VDAG_JR2_Chr3g13180a	Succinate-semialdehyde dehydrogenase	C-compound and carbohydrate metabolism	1.68
VDAG_JR2_Chr4g00210a	Bilirubin oxidase	C-compound and carbohydrate metabolism	6.21
VDAG_JR2_Chr4g00490a	Bifunctional P-450:NADPH-P450 reductase	C-compound and carbohydrate metabolism	2.42
VDAG_JR2_Chr4g01630a	Aerobactin siderophore biosynthesis protein iucB	C-compound and carbohydrate metabolism	3.37
VDAG_JR2_Chr4g03030a	Bifunctional P-450:NADPH-P450 reductase	C-compound and carbohydrate metabolism	1.69
VDAG_JR2_Chr4g03140a	Carnitine O-palmitoyltransferase I	C-compound and carbohydrate metabolism	1.14
VDAG_JR2_Chr4g04660a	Cytochrome b2	C-compound and carbohydrate metabolism	4.01
VDAG_JR2_Chr4g04990a	Phosphoglycerate mutase family protein	C-compound and carbohydrate metabolism	1.82
VDAG_JR2_Chr4g05020a	Lactose regulatory protein LAC9	C-compound and carbohydrate metabolism	1.25
VDAG_JR2_Chr4g05190a	Putative uncharacterized protein	C-compound and carbohydrate metabolism	1.12
VDAG_JR2_Chr4g05940a	Alpha-glucosides permease MPH2/3	C-compound and carbohydrate metabolism	4.31
VDAG_JR2_Chr4g06500a	Proton myo-inositol cotransporter	C-compound and carbohydrate metabolism	1.73
VDAG_JR2_Chr4g07460a	FAD binding domain-containing protein	C-compound and carbohydrate metabolism	1.27
VDAG_JR2_Chr4g08080a	1,3(4)-beta-glucanase	C-compound and carbohydrate metabolism	1.34
VDAG_JR2_Chr4g09320a	Phosphoenolpyruvate carboxykinase	C-compound and carbohydrate metabolism	1.32
VDAG_JR2_Chr4g09630a	Carbonic anhydrase	C-compound and carbohydrate metabolism	2.83
VDAG_JR2_Chr4g10620a	Salicylate hydroxylase	C-compound and carbohydrate metabolism	3.47
VDAG_JR2_Chr4g11100a	Mannan endo-1,4-beta-mannosidase	C-compound and carbohydrate metabolism	1.40
VDAG_JR2_Chr4g11730a	Quinone oxidoreductase	C-compound and carbohydrate metabolism	1.76
VDAG_JR2_Chr4g11890a	Glucanase (EC 3.2.1.-)	C-compound and carbohydrate metabolism	1.66
VDAG_JR2_Chr4g12150a	3-oxoacyl-[acyl-carrier-protein] reductase	C-compound and carbohydrate metabolism	1.26
VDAG_JR2_Chr4g12400a	Oxidoreductase	C-compound and carbohydrate metabolism	3.07

Gene identifier	Protein name	FunCat description	log_2(fold change)
VDAG_JR2_Chr4g12450a	Glucanase B	C-compound and carbohydrate metabolism	3.04
VDAG_JR2_Chr5g00060a	Aldehyde dehydrogenase	C-compound and carbohydrate metabolism	3.15
VDAG_JR2_Chr5g00860a	Glucose oxidase	C-compound and carbohydrate metabolism	1.39
VDAG_JR2_Chr5g03220a	Alpha-galactosidase (EC 3.2.1.22) (Melibiase)	C-compound and carbohydrate metabolism	1.53
VDAG_JR2_Chr5g03240a	Maltose permease MAL31	C-compound and carbohydrate metabolism	1.20
VDAG_JR2_Chr5g04160a	Proton myo-inositol cotransporter	C-compound and carbohydrate metabolism	2.51
VDAG_JR2_Chr5g04280a	NAD(P) transhydrogenase	C-compound and carbohydrate metabolism	1.85
VDAG_JR2_Chr5g04430a	Glutathione-independent formaldehyde dehydrogenase	C-compound and carbohydrate metabolism	2.63
VDAG_JR2_Chr5g06370a	Beta-galactosidase	C-compound and carbohydrate metabolism	1.25
VDAG_JR2_Chr5g07180a	Rhamnogalacturonan acetylesterase	C-compound and carbohydrate metabolism	1.63
VDAG_JR2_Chr5g11040a	Alcohol oxidase	C-compound and carbohydrate metabolism	3.50
VDAG_JR2_Chr5g11880a	Choline dehydrogenase	C-compound and carbohydrate metabolism	1.24
VDAG_JR2_Chr6g01130a	FAD binding domain-containing protein	C-compound and carbohydrate metabolism	5.17
VDAG_JR2_Chr6g02300a	Choline dehydrogenase	C-compound and carbohydrate metabolism	2.31
VDAG_JR2_Chr6g02560a	Putative uncharacterized protein	C-compound and carbohydrate metabolism	1.27
VDAG_JR2_Chr6g02580a	Xylulose-5-phosphate phosphoketolase	C-compound and carbohydrate metabolism	1.20
VDAG_JR2_Chr6g03200a	Aldehyde reductase	C-compound and carbohydrate metabolism	2.87
VDAG_JR2_Chr6g04040a	Endo-1,4-beta-xylanase (EC 3.2.1.8)	C-compound and carbohydrate metabolism	1.42
VDAG_JR2_Chr6g04060a	Beta-fructofuranosidase	C-compound and carbohydrate metabolism	1.28
VDAG_JR2_Chr6g05470a	Short chain dehydrogenase	C-compound and carbohydrate metabolism	1.20
VDAG_JR2_Chr6g06050a	Alcohol dehydrogenase	C-compound and carbohydrate metabolism	2.02
VDAG_JR2_Chr6g06060a	4-trimethylaminobutyraldehyde dehydrogenase	C-compound and carbohydrate metabolism	1.39
VDAG_JR2_Chr6g08420a	Amine oxidase B	C-compound and carbohydrate metabolism	1.26
VDAG_JR2_Chr6g08780a	Alpha-xylosidase	C-compound and carbohydrate metabolism	1.11
VDAG_JR2_Chr6g08800a	General alpha-glucoside permease	C-compound and carbohydrate metabolism	3.19
VDAG_JR2_Chr6g09440a	Aldose 1-epimerase	C-compound and carbohydrate metabolism	1.93

Gene identifier	Protein name	FunCat description	log_2(fold change)
VDAG_JR2_Chr6g09680a	Alcohol oxidase	C-compound and carbohydrate metabolism	1.47
VDAG_JR2_Chr6g10300a	Acetolactate synthase	C-compound and carbohydrate metabolism	2.33
VDAG_JR2_Chr6g10630a	Succinyl-CoA:3-ketoacid-coenzyme A transferase (EC 2.8.3.5)	C-compound and carbohydrate metabolism	1.87
VDAG_JR2_Chr7g00085a	Malic enzyme	C-compound and carbohydrate metabolism	1.94
VDAG_JR2_Chr7g00310a	Glucanase (EC 3.2.1.-)	C-compound and carbohydrate metabolism	1.54
VDAG_JR2_Chr7g01080a	3-hydroxyacyl-CoA dehydrogenase type-2	C-compound and carbohydrate metabolism	1.23
VDAG_JR2_Chr7g02610a	Putative uncharacterized protein	C-compound and carbohydrate metabolism	1.74
VDAG_JR2_Chr7g02640a	Osmotic growth protein	C-compound and carbohydrate metabolism	1.68
VDAG_JR2_Chr7g03350a	Exo-rhamnogalacturonase B	C-compound and carbohydrate metabolism	1.10
VDAG_JR2_Chr7g03620a	Aldehyde dehydrogenase	C-compound and carbohydrate metabolism	1.02
VDAG_JR2_Chr7g05780a	Putative uncharacterized protein	C-compound and carbohydrate metabolism	1.75
VDAG_JR2_Chr7g08570a	Putative uncharacterized protein	C-compound and carbohydrate metabolism	1.10
VDAG_JR2_Chr7g09240a	Pectate lyase A	C-compound and carbohydrate metabolism	1.50
VDAG_JR2_Chr7g09570a	Sugar transporter	C-compound and carbohydrate metabolism	1.01
VDAG_JR2_Chr7g09740a	Epoxide hydrolase	C-compound and carbohydrate metabolism	1.05
VDAG_JR2_Chr8g00100a	Lactonohydrolase	C-compound and carbohydrate metabolism	1.91
VDAG_JR2_Chr8g00620a	Glycerate kinase	C-compound and carbohydrate metabolism	3.13
VDAG_JR2_Chr8g01010a	Endoplasmic reticulum mannosyl-oligosaccharide 1,2-alpha-mannosidase	C-compound and carbohydrate metabolism	1.71
VDAG_JR2_Chr8g01020a	D-lactate dehydrogenase	C-compound and carbohydrate metabolism	1.80
VDAG_JR2_Chr8g01390a	Maltose permease MAL31	C-compound and carbohydrate metabolism	3.28
VDAG_JR2_Chr8g01610a	Maleylacetate reductase	C-compound and carbohydrate metabolism	2.27
VDAG_JR2_Chr8g01840a	Sugar transporter family protein	C-compound and carbohydrate metabolism	1.33
VDAG_JR2_Chr8g02110a	Alcohol dehydrogenase	C-compound and carbohydrate metabolism	1.69
VDAG_JR2_Chr8g02120a	Aldehyde dehydrogenase	C-compound and carbohydrate metabolism	1.11
VDAG_JR2_Chr8g02750a	GS1 protein	C-compound and carbohydrate metabolism	2.92
VDAG_JR2_Chr8g03620a	Alcohol dehydrogenase	C-compound and carbohydrate metabolism	1.79
VDAG_JR2_Chr8g03780a	Pectinesterase	C-compound and carbohydrate metabolism	1.20

Gene identifier	Protein name	FunCat description	log_2(fold change)
VDAG_JR2_Chr8g07660a	Alpha-galactosidase (EC 3.2.1.22) (Melibiase)	C-compound and carbohydrate metabolism	1.15
VDAG_JR2_Chr8g07670a	Maltose permease MAL31	C-compound and carbohydrate metabolism	1.64
VDAG_JR2_Chr8g09210a	Carboxylic acid transport protein	C-compound and carbohydrate metabolism	7.57
VDAG_JR2_Chr8g09450a	4-coumarate-CoA ligase	C-compound and carbohydrate metabolism	1.95
VDAG_JR2_Chr8g09730a	Putative uncharacterized protein	C-compound and carbohydrate metabolism	4.46
VDAG_JR2_Chr1g01070a	Putative uncharacterized protein	C-compound and carbohydrate transport	4.75
VDAG_JR2_Chr1g01080a	Maltose permease	C-compound and carbohydrate transport	2.77
VDAG_JR2_Chr1g01185a	Hexose transporter	C-compound and carbohydrate transport	1.51
VDAG_JR2_Chr1g01440a	Sugar transporter	C-compound and carbohydrate transport	1.62
VDAG_JR2_Chr1g01710a	Inner membrane transport protein yfaV	C-compound and carbohydrate transport	1.52
VDAG_JR2_Chr1g02220a	Sugar transporter	C-compound and carbohydrate transport	2.06
VDAG_JR2_Chr1g05760a	Galactose-proton symporter	C-compound and carbohydrate transport	1.70
VDAG_JR2_Chr1g10200a	Galactose-proton symporter	C-compound and carbohydrate transport	2.85
VDAG_JR2_Chr1g11690a	Putative uncharacterized protein	C-compound and carbohydrate transport	1.34
VDAG_JR2_Chr1g12250a	Choline transport protein	C-compound and carbohydrate transport	1.50
VDAG_JR2_Chr1g18850a	Putative uncharacterized protein	C-compound and carbohydrate transport	1.14
VDAG_JR2_Chr1g22050a	Sugar transporter STL1	C-compound and carbohydrate transport	2.06
VDAG_JR2_Chr1g24520a	Sugar transporter	C-compound and carbohydrate transport	1.70
VDAG_JR2_Chr1g25420a	Urea active transporter	C-compound and carbohydrate transport	1.17
VDAG_JR2_Chr1g26920a	High-affinity glucose transporter SNF3	C-compound and carbohydrate transport	1.27
VDAG_JR2_Chr1g27550a	Urea active transporter	C-compound and carbohydrate transport	2.12
VDAG_JR2_Chr2g02540a	Pantothenate transporter liz1	C-compound and carbohydrate transport	7.40
VDAG_JR2_Chr2g02970a	Integral membrane protein	C-compound and carbohydrate transport	1.18
VDAG_JR2_Chr2g04380a	Integral membrane protein	C-compound and carbohydrate transport	1.88
VDAG_JR2_Chr2g04500a	Quinate permease	C-compound and carbohydrate transport	4.34
VDAG_JR2_Chr2g08880a	Maltose permease MAL31	C-compound and carbohydrate transport	1.34
VDAG_JR2_Chr2g12320a	General alpha-glucoside permease	C-compound and carbohydrate transport	1.43
VDAG_JR2_Chr2g13170a	Putative uncharacterized protein	C-compound and carbohydrate transport	1.08

Gene identifier	Protein name	FunCat description	log_2(fold change)
VDAG_JR2_Chr3g01050a	Membrane transporter D1	C-compound and carbohydrate transport	1.34
VDAG_JR2_Chr3g10430a	Lactose permease	C-compound and carbohydrate transport	4.55
VDAG_JR2_Chr3g10590a	General alpha-glucoside permease	C-compound and carbohydrate transport	1.66
VDAG_JR2_Chr3g12560a	Maltose permease MAL31	C-compound and carbohydrate transport	1.21
VDAG_JR2_Chr3g12800a	Putative uncharacterized protein	C-compound and carbohydrate transport	1.71
VDAG_JR2_Chr3g12900a	Alpha-glucosides permease MPH2/3	C-compound and carbohydrate transport	1.08
VDAG_JR2_Chr4g01150a	Glucose/galactose transporter	C-compound and carbohydrate transport	1.91
VDAG_JR2_Chr4g01750a	Sugar transporter	C-compound and carbohydrate transport	1.33
VDAG_JR2_Chr4g02440a	Putative uncharacterized protein	C-compound and carbohydrate transport	1.34
VDAG_JR2_Chr4g03140a	Carnitine O-palmitoyltransferase I	C-compound and carbohydrate transport	1.14
VDAG_JR2_Chr4g05930a	Putative uncharacterized protein	C-compound and carbohydrate transport	3.10
VDAG_JR2_Chr4g05940a	Alpha-glucosides permease MPH2/3	C-compound and carbohydrate transport	4.31
VDAG_JR2_Chr4g06500a	Proton myo-inositol cotransporter	C-compound and carbohydrate transport	1.73
VDAG_JR2_Chr4g10760a	Choline transport protein	C-compound and carbohydrate transport	4.55
VDAG_JR2_Chr4g11930a	Integral membrane protein	C-compound and carbohydrate transport	1.68
VDAG_JR2_Chr4g12110a	High-affinity nicotinic acid transporter	C-compound and carbohydrate transport	1.50
VDAG_JR2_Chr5g00190a	Glucose transporter	C-compound and carbohydrate transport	1.09
VDAG_JR2_Chr5g03240a	Maltose permease MAL31	C-compound and carbohydrate transport	1.20
VDAG_JR2_Chr5g03740a	Lactose permease	C-compound and carbohydrate transport	1.89
VDAG_JR2_Chr5g04160a	Proton myo-inositol cotransporter	C-compound and carbohydrate transport	2.51
VDAG_JR2_Chr5g05100a	Quinate permease	C-compound and carbohydrate transport	1.56
VDAG_JR2_Chr5g06010a	DUF250 domain membrane protein	C-compound and carbohydrate transport	1.31
VDAG_JR2_Chr5g06930a	Choline transport protein	C-compound and carbohydrate transport	4.08
VDAG_JR2_Chr5g07790a	Solute carrier family 35 member E3	C-compound and carbohydrate transport	1.61
VDAG_JR2_Chr6g01260a	Pantothenate transporter liz1	C-compound and carbohydrate transport	2.12
VDAG_JR2_Chr6g01290a	Lactose permease	C-compound and carbohydrate transport	1.17
VDAG_JR2_Chr6g08800a	General alpha-glucoside permease	C-compound and carbohydrate transport	3.19
VDAG_JR2_Chr6g09100a	Putative uncharacterized protein	C-compound and carbohydrate transport	1.69
VDAG_JR2_Chr6g09710a	Quinate permease	C-compound and carbohydrate transport	1.04

Gene identifier	Protein name	FunCat description	$\log_2$(fold change)
VDAG_JR2_Chr6g10740a	High-affinity glucose transporter RGT2	C-compound and carbohydrate transport	6.81
VDAG_JR2_Chr7g00030a	Pantothenate transporter liz1	C-compound and carbohydrate transport	1.02
VDAG_JR2_Chr7g03610a	Riboflavin transporter MCH5	C-compound and carbohydrate transport	2.36
VDAG_JR2_Chr7g07610a	Choline transport protein	C-compound and carbohydrate transport	1.12
VDAG_JR2_Chr7g09570a	Sugar transporter	C-compound and carbohydrate transport	1.01
VDAG_JR2_Chr8g00880a	Lactose permease	C-compound and carbohydrate transport	1.88
VDAG_JR2_Chr8g00990a	Sugar transporter	C-compound and carbohydrate transport	2.49
VDAG_JR2_Chr8g01050a	Phthalate transporter	C-compound and carbohydrate transport	1.36
VDAG_JR2_Chr8g01260a	L-fucose transporter	C-compound and carbohydrate transport	5.47
VDAG_JR2_Chr8g01390a	Maltose permease MAL31	C-compound and carbohydrate transport	3.28
VDAG_JR2_Chr8g01840a	Sugar transporter family protein	C-compound and carbohydrate transport	1.33
VDAG_JR2_Chr8g02900a	Putative uncharacterized protein	C-compound and carbohydrate transport	2.18
VDAG_JR2_Chr8g07670a	Maltose permease MAL31	C-compound and carbohydrate transport	1.64
VDAG_JR2_Chr8g09210a	Carboxylic acid transport protein	C-compound and carbohydrate transport	7.57
VDAG_JR2_Chr8g11120a	Integral membrane protein	C-compound and carbohydrate transport	2.51
VDAG_JR2_Chr1g00390a	Iron transport multicopper oxidase FET3	Cellular import	4.06
VDAG_JR2_Chr1g01070a	Putative uncharacterized protein	Cellular import	4.75
VDAG_JR2_Chr1g01185a	Hexose transporter	Cellular import	1.51
VDAG_JR2_Chr1g01440a	Sugar transporter	Cellular import	1.62
VDAG_JR2_Chr1g01710a	Inner membrane transport protein yfaV	Cellular import	1.52
VDAG_JR2_Chr1g02220a	Sugar transporter	Cellular import	2.06
VDAG_JR2_Chr1g05010a	Peptide transporter PTR2	Cellular import	3.23
VDAG_JR2_Chr1g05030a	Ferric-chelate reductase	Cellular import	1.93
VDAG_JR2_Chr1g05760a	Galactose-proton symporter	Cellular import	1.70
VDAG_JR2_Chr1g08890a	Putative uncharacterized protein	Cellular import	1.29
VDAG_JR2_Chr1g10200a	Galactose-proton symporter	Cellular import	2.85
VDAG_JR2_Chr1g12250a	Choline transport protein	Cellular import	1.50
VDAG_JR2_Chr1g15800a	Cobalt uptake protein COT1	Cellular import	2.19
VDAG_JR2_Chr1g22050a	Sugar transporter STL1	Cellular import	2.06

Gene identifier	Protein name	FunCat description	log_2(fold change)
VDAG_JR2_Chr1g24150a	Uracil permease	Cellular import	4.12
VDAG_JR2_Chr1g24520a	Sugar transporter	Cellular import	1.70
VDAG_JR2_Chr1g24720a	Allantoate permease	Cellular import	1.03
VDAG_JR2_Chr1g26920a	High-affinity glucose transporter SNF3	Cellular import	1.27
VDAG_JR2_Chr2g02900a	High-affinity methionine permease	Cellular import	1.66
VDAG_JR2_Chr2g03860a	Inner membrane transport protein yfaV	Cellular import	2.02
VDAG_JR2_Chr2g04500a	Quinate permease	Cellular import	4.34
VDAG_JR2_Chr2g04660a	Lysine-specific permease	Cellular import	1.42
VDAG_JR2_Chr2g08730a	Putative uncharacterized protein	Cellular import	1.43
VDAG_JR2_Chr2g08880a	Maltose permease MAL31	Cellular import	1.34
VDAG_JR2_Chr2g09810a	Inner membrane protein yicO	Cellular import	1.44
VDAG_JR2_Chr2g12320a	General alpha-glucoside permease	Cellular import	1.43
VDAG_JR2_Chr2g13170a	Putative uncharacterized protein	Cellular import	1.08
VDAG_JR2_Chr3g01050a	Membrane transporter D1	Cellular import	1.34
VDAG_JR2_Chr3g10430a	Lactose permease	Cellular import	4.55
VDAG_JR2_Chr3g12560a	Maltose permease MAL31	Cellular import	1.21
VDAG_JR2_Chr3g12900a	Alpha-glucosides permease MPH2/3	Cellular import	1.08
VDAG_JR2_Chr4g00720a	Phosphate transporter family protein	Cellular import	1.67
VDAG_JR2_Chr4g01150a	Glucose/galactose transporter	Cellular import	1.91
VDAG_JR2_Chr4g01620a	Sulfate permease	Cellular import	3.70
VDAG_JR2_Chr4g01750a	Sugar transporter	Cellular import	1.33
VDAG_JR2_Chr4g06500a	Proton myo-inositol cotransporter	Cellular import	1.73
VDAG_JR2_Chr4g07840a	Potassium transporter 1	Cellular import	1.47
VDAG_JR2_Chr4g10760a	Choline transport protein	Cellular import	4.55
VDAG_JR2_Chr4g11570a	Metalloreductase	Cellular import	1.15
VDAG_JR2_Chr4g11820a	General amino-acid permease GAP1	Cellular import	2.99
VDAG_JR2_Chr4g12160a	Ammonium transporter 1	Cellular import	2.22
VDAG_JR2_Chr4g12390a	Putative uncharacterized protein	Cellular import	8.31
VDAG_JR2_Chr5g00190a	Glucose transporter	Cellular import	1.09

Gene identifier	Protein name	FunCat description	log_2(fold change)
VDAG_JR2_Chr5g01900a	High-affinity potassium transport protein	Cellular import	3.47
VDAG_JR2_Chr5g03240a	Maltose permease MAL31	Cellular import	1.20
VDAG_JR2_Chr5g03740a	Lactose permease	Cellular import	1.89
VDAG_JR2_Chr5g06930a	Choline transport protein	Cellular import	4.08
VDAG_JR2_Chr6g01260a	Pantothenate transporter liz1	Cellular import	2.12
VDAG_JR2_Chr6g01290a	Lactose permease	Cellular import	1.17
VDAG_JR2_Chr6g02110a	Thiamine transporter	Cellular import	1.23
VDAG_JR2_Chr6g02450a	Putative uncharacterized protein	Cellular import	1.17
VDAG_JR2_Chr6g03600a	Low-affinity potassium transport protein	Cellular import	1.13
VDAG_JR2_Chr6g04250a	Pantothenate transporter liz1	Cellular import	3.63
VDAG_JR2_Chr6g07520a	Ferric reductase transmembrane component 5	Cellular import	1.22
VDAG_JR2_Chr6g08800a	General alpha-glucoside permease	Cellular import	3.19
VDAG_JR2_Chr6g09380a	Allantoate permease	Cellular import	1.29
VDAG_JR2_Chr6g10740a	High-affinity glucose transporter RGT2	Cellular import	6.81
VDAG_JR2_Chr7g00030a	Pantothenate transporter liz1	Cellular import	1.02
VDAG_JR2_Chr7g02750a	Low-affinity potassium transport protein	Cellular import	1.50
VDAG_JR2_Chr7g03220a	Allantoate permease	Cellular import	1.78
VDAG_JR2_Chr7g05140a	Uric acid-xanthine permease	Cellular import	1.33
VDAG_JR2_Chr7g05460a	Putative uncharacterized protein	Cellular import	1.22
VDAG_JR2_Chr7g06430a	FreB	Cellular import	1.69
VDAG_JR2_Chr7g07610a	Choline transport protein	Cellular import	1.12
VDAG_JR2_Chr7g10310a	Amino-acid permease inda1	Cellular import	1.13
VDAG_JR2_Chr7g10440a	Sodium/nucleoside cotransporter	Cellular import	1.21
VDAG_JR2_Chr8g00880a	Lactose permease	Cellular import	1.88
VDAG_JR2_Chr8g00990a	Sugar transporter	Cellular import	2.49
VDAG_JR2_Chr8g01260a	L-fucose transporter	Cellular import	5.47
VDAG_JR2_Chr8g01390a	Maltose permease MAL31	Cellular import	3.28
VDAG_JR2_Chr8g02900a	Putative uncharacterized protein	Cellular import	2.18

Gene identifier	Protein name	FunCat description	log_2(fold change)
VDAG_JR2_Chr8g06660a	Ammonium transporter MEP1	Cellular import	1.29
VDAG_JR2_Chr8g07670a	Maltose permease MAL31	Cellular import	1.64
VDAG_JR2_Chr8g10350a	Phthalate transporter	Cellular import	3.85
VDAG_JR2_Chr8g11110a	Allantoate permease	Cellular import	1.96
VDAG_JR2_Chr1g00390a	Iron transport multicopper oxidase FET3	Homeostasis of metal ions (Na, K, Ca etc.)	4.06
VDAG_JR2_Chr1g00400a	Plasma membrane iron permease	Homeostasis of metal ions (Na, K, Ca etc.)	2.03
VDAG_JR2_Chr1g02790a	Calcium-transporting ATPase	Homeostasis of metal ions (Na, K, Ca etc.)	1.45
VDAG_JR2_Chr1g05030a	Ferric-chelate reductase	Homeostasis of metal ions (Na, K, Ca etc.)	1.93
VDAG_JR2_Chr1g08890a	Putative uncharacterized protein	Homeostasis of metal ions (Na, K, Ca etc.)	1.29
VDAG_JR2_Chr1g15800a	Cobalt uptake protein COT1	Homeostasis of metal ions (Na, K, Ca etc.)	2.19
VDAG_JR2_Chr1g15850a	ABC transporter CDR4	Homeostasis of metal ions (Na, K, Ca etc.)	1.22
VDAG_JR2_Chr1g18920a	Putative uncharacterized protein	Homeostasis of metal ions (Na, K, Ca etc.)	1.06
VDAG_JR2_Chr1g22590a	Vacuolar calcium ion transporter	Homeostasis of metal ions (Na, K, Ca etc.)	3.81
VDAG_JR2_Chr1g26890a	Pleiotropic ABC multiple drug efflux transporter	Homeostasis of metal ions (Na, K, Ca etc.)	1.07
VDAG_JR2_Chr1g29160a	Calcium-transporting ATPase	Homeostasis of metal ions (Na, K, Ca etc.)	1.22
VDAG_JR2_Chr2g02790a	Glutamate carboxypeptidase	Homeostasis of metal ions (Na, K, Ca etc.)	1.07
VDAG_JR2_Chr2g04730a	Zinc/iron transporter protein	Homeostasis of metal ions (Na, K, Ca etc.)	1.13
VDAG_JR2_Chr2g13190a	Voltage-gated potassium channel subunit beta-3	Homeostasis of metal ions (Na, K, Ca etc.)	1.20
VDAG_JR2_Chr3g05190a	Sodium/calcium exchanger protein	Homeostasis of metal ions (Na, K, Ca etc.)	1.17
VDAG_JR2_Chr3g11520a	Potassium channel	Homeostasis of metal ions (Na, K, Ca etc.)	1.06
VDAG_JR2_Chr4g00520a	Nonribosomal peptide synthetase	Homeostasis of metal ions (Na, K, Ca etc.)	1.15
VDAG_JR2_Chr4g01640a	DUF907 domain-containing protein	Homeostasis of metal ions (Na, K, Ca etc.)	4.68
VDAG_JR2_Chr4g04200a	Calcium permease	Homeostasis of metal ions (Na, K, Ca etc.)	1.15
VDAG_JR2_Chr4g11570a	Metalloreductase	Homeostasis of metal ions (Na, K, Ca etc.)	1.15
VDAG_JR2_Chr4g11690a	Plasma membrane iron permease	Homeostasis of metal ions (Na, K, Ca etc.)	1.05
VDAG_JR2_Chr4g11820a	General amino-acid permease GAP1	Homeostasis of metal ions (Na, K, Ca etc.)	2.99
VDAG_JR2_Chr5g01900a	High-affinity potassium transport protein	Homeostasis of metal ions (Na, K, Ca etc.)	3.47

Gene identifier	Protein name	FunCat description	log_2(fold change)
VDAG_JR2_Chr5g01910a	Na+/H+ antiporter	Homeostasis of metal ions (Na, K, Ca etc.)	2.53
VDAG_JR2_Chr5g10400a	Calcium-channel protein cch1	Homeostasis of metal ions (Na, K, Ca etc.)	1.30
VDAG_JR2_Chr5g12230a	Na+/H+ antiporter	Homeostasis of metal ions (Na, K, Ca etc.)	1.49
VDAG_JR2_Chr6g02450a	Putative uncharacterized protein	Homeostasis of metal ions (Na, K, Ca etc.)	1.17
VDAG_JR2_Chr6g03320a	Putative uncharacterized protein	Homeostasis of metal ions (Na, K, Ca etc.)	1.06
VDAG_JR2_Chr6g03600a	Low-affinity potassium transport protein	Homeostasis of metal ions (Na, K, Ca etc.)	1.13
VDAG_JR2_Chr6g04210a	Siderophore iron transporter mirB	Homeostasis of metal ions (Na, K, Ca etc.)	1.70
VDAG_JR2_Chr6g04520a	Cell division control protein	Homeostasis of metal ions (Na, K, Ca etc.)	1.20
VDAG_JR2_Chr6g06420a	Multidrug resistance protein CDR2	Homeostasis of metal ions (Na, K, Ca etc.)	1.11
VDAG_JR2_Chr6g07520a	Ferric reductase transmembrane component 5	Homeostasis of metal ions (Na, K, Ca etc.)	1.22
VDAG_JR2_Chr6g09800a	K+/H+ antiporter 1	Homeostasis of metal ions (Na, K, Ca etc.)	2.20
VDAG_JR2_Chr7g02750a	Low-affinity potassium transport protein	Homeostasis of metal ions (Na, K, Ca etc.)	1.50
VDAG_JR2_Chr7g05460a	Putative uncharacterized protein	Homeostasis of metal ions (Na, K, Ca etc.)	1.22
VDAG_JR2_Chr7g06430a	FreB	Homeostasis of metal ions (Na, K, Ca etc.)	1.69
VDAG_JR2_Chr7g10310a	Amino-acid permease inda1	Homeostasis of metal ions (Na, K, Ca etc.)	1.13
VDAG_JR2_Chr8g10170a	Siderophore iron transporter mirB	Homeostasis of metal ions (Na, K, Ca etc.)	2.25
VDAG_JR2_Chr8g10450a	Annexin-B12	Homeostasis of metal ions (Na, K, Ca etc.)	2.63
VDAG_JR2_Chr1g10700a	Solute carrier family 12 member 6	Ion transport	2.26
VDAG_JR2_Chr1g15800a	Cobalt uptake protein COT1	Ion transport	2.19
VDAG_JR2_Chr1g24720a	Allantoate permease	Ion transport	1.03
VDAG_JR2_Chr1g29160a	Calcium-transporting ATPase	Ion transport	1.22
VDAG_JR2_Chr2g07940a	Phospholipid-transporting ATPase	Ion transport	1.15
VDAG_JR2_Chr2g13190a	Voltage-gated potassium channel subunit beta-3	Ion transport	1.20
VDAG_JR2_Chr3g05190a	Sodium/calcium exchanger protein	Ion transport	1.17
VDAG_JR2_Chr3g11010a	Vacuolar ATP synthase 98 kDa subunit	Ion transport	2.69
VDAG_JR2_Chr5g01900a	High-affinity potassium transport protein	Ion transport	3.47
VDAG_JR2_Chr5g05790a	Membrane transporter	Ion transport	1.24

**Gene identifier**	**Protein name**	**FunCat description**	**log_2(fold change)**
VDAG_JR2_Chr5g10400a	Calcium-channel protein cch1	Ion transport	1.30
VDAG_JR2_Chr6g03600a	Low-affinity potassium transport protein	Ion transport	1.13
VDAG_JR2_Chr6g09380a	Allantoate permease	Ion transport	1.29
VDAG_JR2_Chr6g09800a	K+/H+ antiporter 1	Ion transport	2.20
VDAG_JR2_Chr7g02750a	Low-affinity potassium transport protein	Ion transport	1.50
VDAG_JR2_Chr7g07860a	Chloride channel protein	Ion transport	1.31
VDAG_JR2_Chr8g09730a	Putative uncharacterized protein	Ion transport	4.46
VDAG_JR2_Chr1g15230a	Spermidine synthase	Metabolism of amines	1.58
VDAG_JR2_Chr2g13240a	Choline dehydrogenase	Metabolism of amines	4.55
VDAG_JR2_Chr3g12360a	Cellobiose dehydrogenase	Metabolism of amines	2.27
VDAG_JR2_Chr4g10210a	Amine oxidase	Metabolism of amines	1.40
VDAG_JR2_Chr4g10770a	Amine oxidase	Metabolism of amines	3.27
VDAG_JR2_Chr5g00860a	Glucose oxidase	Metabolism of amines	1.39
VDAG_JR2_Chr5g00970a	Choline monooxygenase	Metabolism of amines	1.93
VDAG_JR2_Chr5g06940a	Monoamine oxidase N	Metabolism of amines	4.95
VDAG_JR2_Chr5g11040a	Alcohol oxidase	Metabolism of amines	3.50
VDAG_JR2_Chr5g11880a	Choline dehydrogenase	Metabolism of amines	1.24
VDAG_JR2_Chr6g02300a	Choline dehydrogenase	Metabolism of amines	2.31
VDAG_JR2_Chr6g08420a	Amine oxidase B	Metabolism of amines	1.26
VDAG_JR2_Chr6g09680a	Alcohol oxidase	Metabolism of amines	1.47
VDAG_JR2_Chr7g07620a	Amine oxidase	Metabolism of amines	2.22
VDAG_JR2_Chr1g01070a	Putative uncharacterized protein	Non-vesicular cellular import	4.75
VDAG_JR2_Chr1g01080a	Maltose permease	Non-vesicular cellular import	2.77
VDAG_JR2_Chr1g01185a	Hexose transporter	Non-vesicular cellular import	1.51
VDAG_JR2_Chr1g01440a	Sugar transporter	Non-vesicular cellular import	1.62
VDAG_JR2_Chr1g01710a	Inner membrane transport protein yfaV	Non-vesicular cellular import	1.52
VDAG_JR2_Chr1g02220a	Sugar transporter	Non-vesicular cellular import	2.06
VDAG_JR2_Chr1g05010a	Peptide transporter PTR2	Non-vesicular cellular import	3.23

Gene identifier	Protein name	FunCat description	log_2(fold change)
VDAG_JR2_Chr1g05760a	Galactose-proton symporter	Non-vesicular cellular import	1.70
VDAG_JR2_Chr1g10200a	Galactose-proton symporter	Non-vesicular cellular import	2.85
VDAG_JR2_Chr1g18850a	Putative uncharacterized protein	Non-vesicular cellular import	1.14
VDAG_JR2_Chr1g22050a	Sugar transporter STL1	Non-vesicular cellular import	2.06
VDAG_JR2_Chr1g23370a	Aquaporin-2	Non-vesicular cellular import	3.31
VDAG_JR2_Chr1g24150a	Uracil permease	Non-vesicular cellular import	4.12
VDAG_JR2_Chr1g24520a	Sugar transporter	Non-vesicular cellular import	1.70
VDAG_JR2_Chr1g24720a	Allantoate permease	Non-vesicular cellular import	1.03
VDAG_JR2_Chr1g24790a	High-affinity nicotinic acid transporter	Non-vesicular cellular import	2.54
VDAG_JR2_Chr1g26920a	High-affinity glucose transporter SNF3	Non-vesicular cellular import	1.27
VDAG_JR2_Chr2g02900a	High-affinity methionine permease	Non-vesicular cellular import	1.66
VDAG_JR2_Chr2g03860a	Inner membrane transport protein yfaV	Non-vesicular cellular import	2.02
VDAG_JR2_Chr2g04500a	Quinate permease	Non-vesicular cellular import	4.34
VDAG_JR2_Chr2g04660a	Lysine-specific permease	Non-vesicular cellular import	1.42
VDAG_JR2_Chr2g04730a	Zinc/iron transporter protein	Non-vesicular cellular import	1.13
VDAG_JR2_Chr2g08730a	Putative uncharacterized protein	Non-vesicular cellular import	1.43
VDAG_JR2_Chr2g08880a	Maltose permease MAL31	Non-vesicular cellular import	1.34
VDAG_JR2_Chr2g12320a	General alpha-glucoside permease	Non-vesicular cellular import	1.43
VDAG_JR2_Chr2g13170a	Putative uncharacterized protein	Non-vesicular cellular import	1.08
VDAG_JR2_Chr3g01050a	Membrane transporter D1	Non-vesicular cellular import	1.34
VDAG_JR2_Chr3g10430a	Lactose permease	Non-vesicular cellular import	4.55
VDAG_JR2_Chr3g10590a	General alpha-glucoside permease	Non-vesicular cellular import	1.66
VDAG_JR2_Chr3g12560a	Maltose permease MAL31	Non-vesicular cellular import	1.21
VDAG_JR2_Chr3g12900a	Alpha-glucosides permease MPH2/3	Non-vesicular cellular import	1.08
VDAG_JR2_Chr4g01620a	Sulfate permease	Non-vesicular cellular import	3.70
VDAG_JR2_Chr4g01750a	Sugar transporter	Non-vesicular cellular import	1.33
VDAG_JR2_Chr4g01780a	Aquaporin-1	Non-vesicular cellular import	1.14
VDAG_JR2_Chr4g04720a	Proline-specific permease	Non-vesicular cellular import	1.18
VDAG_JR2_Chr4g05940a	Alpha-glucosides permease MPH2/3	Non-vesicular cellular import	4.31

Gene identifier	Protein name	FunCat description	log_2(fold change)
VDAG_JR2_Chr4g06500a	Proton myo-inositol cotransporter	Non-vesicular cellular import	1.73
VDAG_JR2_Chr4g10900a	Vitamin H transporter	Non-vesicular cellular import	3.04
VDAG_JR2_Chr4g11820a	General amino-acid permease GAP1	Non-vesicular cellular import	2.99
VDAG_JR2_Chr4g12110a	High-affinity nicotinic acid transporter	Non-vesicular cellular import	1.50
VDAG_JR2_Chr4g12390a	Putative uncharacterized protein	Non-vesicular cellular import	8.31
VDAG_JR2_Chr4g12420a	Dicarboxylic amino acid permease	Non-vesicular cellular import	2.70
VDAG_JR2_Chr5g00190a	Glucose transporter	Non-vesicular cellular import	1.09
VDAG_JR2_Chr5g01900a	High-affinity potassium transport protein	Non-vesicular cellular import	3.47
VDAG_JR2_Chr5g03240a	Maltose permease MAL31	Non-vesicular cellular import	1.20
VDAG_JR2_Chr5g03740a	Lactose permease	Non-vesicular cellular import	1.89
VDAG_JR2_Chr5g04160a	Proton myo-inositol cotransporter	Non-vesicular cellular import	2.51
VDAG_JR2_Chr5g05100a	Quinate permease	Non-vesicular cellular import	1.56
VDAG_JR2_Chr6g01290a	Lactose permease	Non-vesicular cellular import	1.17
VDAG_JR2_Chr6g02110a	Thiamine transporter	Non-vesicular cellular import	1.23
VDAG_JR2_Chr6g03600a	Low-affinity potassium transport protein	Non-vesicular cellular import	1.13
VDAG_JR2_Chr6g04210a	Siderophore iron transporter mirB	Non-vesicular cellular import	1.70
VDAG_JR2_Chr6g04250a	Pantothenate transporter liz1	Non-vesicular cellular import	3.63
VDAG_JR2_Chr6g08800a	General alpha-glucoside permease	Non-vesicular cellular import	3.19
VDAG_JR2_Chr6g09100a	Putative uncharacterized protein	Non-vesicular cellular import	1.69
VDAG_JR2_Chr6g09380a	Allantoate permease	Non-vesicular cellular import	1.29
VDAG_JR2_Chr6g09710a	Quinate permease	Non-vesicular cellular import	1.04
VDAG_JR2_Chr6g10740a	High-affinity glucose transporter RGT2	Non-vesicular cellular import	6.81
VDAG_JR2_Chr7g02750a	Low-affinity potassium transport protein	Non-vesicular cellular import	1.50
VDAG_JR2_Chr7g03220a	Allantoate permease	Non-vesicular cellular import	1.78
VDAG_JR2_Chr7g03610a	Riboflavin transporter MCH5	Non-vesicular cellular import	2.36
VDAG_JR2_Chr7g05140a	Uric acid-xanthine permease	Non-vesicular cellular import	1.33
VDAG_JR2_Chr7g09570a	Sugar transporter	Non-vesicular cellular import	1.01
VDAG_JR2_Chr7g10310a	Amino-acid permease inda1	Non-vesicular cellular import	1.13
VDAG_JR2_Chr8g00880a	Lactose permease	Non-vesicular cellular import	1.88

Gene identifier	Protein name	FunCat description	log_2(fold change)
VDAG_JR2_Chr8g00990a	Sugar transporter	Non-vesicular cellular import	2.49
VDAG_JR2_Chr8g01050a	Phthalate transporter	Non-vesicular cellular import	1.36
VDAG_JR2_Chr8g01390a	Maltose permease MAL31	Non-vesicular cellular import	3.28
VDAG_JR2_Chr8g02280a	Vitamin H transporter 1	Non-vesicular cellular import	1.44
VDAG_JR2_Chr8g02900a	Putative uncharacterized protein	Non-vesicular cellular import	2.18
VDAG_JR2_Chr8g07670a	Maltose permease MAL31	Non-vesicular cellular import	1.64
VDAG_JR2_Chr8g10170a	Siderophore iron transporter mirB	Non-vesicular cellular import	2.25
VDAG_JR2_Chr8g11110a	Allantoate permease	Non-vesicular cellular import	1.96
VDAG_JR2_Chr1g02780a	Endoglucanase-5	Polysaccharide metabolism	2.14
VDAG_JR2_Chr1g06240a	Beta-glucosidase	Polysaccharide metabolism	4.43
VDAG_JR2_Chr1g06940a	Beta-glucanase	Polysaccharide metabolism	1.36
VDAG_JR2_Chr1g17290a	Alpha-galactosidase (EC 3.2.1.22) (Melibiase)	Polysaccharide metabolism	1.10
VDAG_JR2_Chr1g18230a	Glucoamylase (EC 3.2.1.3) (1,4-alpha-D-glucan glucohydrolase) (Glucan 1,4-alpha-glucosidase)	Polysaccharide metabolism	1.40
VDAG_JR2_Chr1g21910a	Glucanase (EC 3.2.1.-)	Polysaccharide metabolism	3.79
VDAG_JR2_Chr1g22490a	Acetylxylan esterase	Polysaccharide metabolism	1.39
VDAG_JR2_Chr1g22690a	Endo alpha-1,4 polygalactosaminidase precusor	Polysaccharide metabolism	1.18
VDAG_JR2_Chr1g22740a	Endoglucanase-4	Polysaccharide metabolism	1.39
VDAG_JR2_Chr1g24130a	Polygalacturonase	Polysaccharide metabolism	1.98
VDAG_JR2_Chr1g25020a	Glucanase (EC 3.2.1.-)	Polysaccharide metabolism	1.04
VDAG_JR2_Chr1g25430a	Acetylxylan esterase	Polysaccharide metabolism	2.29
VDAG_JR2_Chr1g26170a	Putative uncharacterized protein	Polysaccharide metabolism	1.22
VDAG_JR2_Chr1g27290a	Beta-galactosidase	Polysaccharide metabolism	1.44
VDAG_JR2_Chr1g28060a	Glucan 1,3-beta-glucosidase	Polysaccharide metabolism	2.38
VDAG_JR2_Chr1g28970a	Alpha-galactosidase (EC 3.2.1.22) (Melibiase)	Polysaccharide metabolism	2.20

Gene identifier	Protein name	FunCat description	log_2(fold change)
VDAG_JR2_Chr1g29680a	Glucan 1,3-beta-glucosidase	Polysaccharide metabolism	1.95
VDAG_JR2_Chr2g00430a	Pectate lyase	Polysaccharide metabolism	2.89
VDAG_JR2_Chr2g00660a	Glucanase (EC 3.2.1.-)	Polysaccharide metabolism	1.67
VDAG_JR2_Chr2g00910a	Beta-galactosidase	Polysaccharide metabolism	1.68
VDAG_JR2_Chr2g01760a	Putative uncharacterized protein	Polysaccharide metabolism	1.44
VDAG_JR2_Chr2g02280a	Chitin deacetylase	Polysaccharide metabolism	1.07
VDAG_JR2_Chr2g05870a	Glucoamylase (EC 3.2.1.3) (1,4-alpha-D-glucan glucohydrolase) (Glucan 1,4-alpha-glucosidase)	Polysaccharide metabolism	1.49
VDAG_JR2_Chr2g06830a	Xylosidase/arabinosidase	Polysaccharide metabolism	1.04
VDAG_JR2_Chr2g13290a	Pectate lyase D	Polysaccharide metabolism	1.97
VDAG_JR2_Chr3g02150a	Chitinase	Polysaccharide metabolism	1.17
VDAG_JR2_Chr3g03460a	Alpha-mannosidase	Polysaccharide metabolism	1.12
VDAG_JR2_Chr3g05640a	Glucan endo-1,3-alpha-glucosidase agn1	Polysaccharide metabolism	1.15
VDAG_JR2_Chr3g10320a	Beta-hexosaminidase	Polysaccharide metabolism	1.47
VDAG_JR2_Chr3g10580a	Glycosyl hydrolases family 32 superfamily	Polysaccharide metabolism	2.43
VDAG_JR2_Chr3g12270a	Chitin synthase	Polysaccharide metabolism	1.62
VDAG_JR2_Chr3g12280a	UDP-glucose 6-dehydrogenase (EC 1.1.1.22)	Polysaccharide metabolism	1.18
VDAG_JR2_Chr3g12300a	Pectate lyase	Polysaccharide metabolism	2.07
VDAG_JR2_Chr3g12360a	Cellobiose dehydrogenase	Polysaccharide metabolism	2.27
VDAG_JR2_Chr4g00350a	Polysaccharide deacetylase family protein	Polysaccharide metabolism	1.13
VDAG_JR2_Chr4g11100a	Mannan endo-1,4-beta-mannosidase	Polysaccharide metabolism	1.40
VDAG_JR2_Chr4g11890a	Glucanase (EC 3.2.1.-)	Polysaccharide metabolism	1.66
VDAG_JR2_Chr5g03220a	Alpha-galactosidase (EC 3.2.1.22) (Melibiase)	Polysaccharide metabolism	1.53
VDAG_JR2_Chr5g04120a	Cel74a	Polysaccharide metabolism	1.76
VDAG_JR2_Chr5g04180a	Thermostable beta-glucosidase B	Polysaccharide metabolism	2.48
VDAG_JR2_Chr5g05890a	Glycosyl hydrolase family 43 protein	Polysaccharide metabolism	2.37

Gene identifier	Protein name	FunCat description	$\log_2$(fold change)
VDAG_JR2_Chr5g06020a	Exostosin-2	Polysaccharide metabolism	1.25
VDAG_JR2_Chr5g09380a	Endoglucanase II	Polysaccharide metabolism	4.18
VDAG_JR2_Chr5g09890a	Chitin deacetylase	Polysaccharide metabolism	1.58
VDAG_JR2_Chr6g00450a	Glucan 1,3-beta-glucosidase	Polysaccharide metabolism	1.83
VDAG_JR2_Chr6g04060a	Beta-fructofuranosidase	Polysaccharide metabolism	1.28
VDAG_JR2_Chr6g10890a	Putative uncharacterized protein	Polysaccharide metabolism	2.46
VDAG_JR2_Chr7g00075a	Beta-glucosidase	Polysaccharide metabolism	5.24
VDAG_JR2_Chr7g00310a	Glucanase (EC 3.2.1.-)	Polysaccharide metabolism	1.54
VDAG_JR2_Chr7g01140a	Beta-xylosidase	Polysaccharide metabolism	1.89
VDAG_JR2_Chr7g02610a	Putative uncharacterized protein	Polysaccharide metabolism	1.74
VDAG_JR2_Chr7g03350a	Exo-rhamnogalacturonase B	Polysaccharide metabolism	1.10
VDAG_JR2_Chr7g03570a	Cellobiose dehydrogenase	Polysaccharide metabolism	1.03
VDAG_JR2_Chr7g05780a	Putative uncharacterized protein	Polysaccharide metabolism	1.75
VDAG_JR2_Chr7g09240a	Pectate lyase A	Polysaccharide metabolism	1.50
VDAG_JR2_Chr8g00600a	Thermostable beta-glucosidase B	Polysaccharide metabolism	1.03
VDAG_JR2_Chr8g01010a	Endoplasmic reticulum mannosyl-oligosaccharide 1,2-alpha-mannosidase	Polysaccharide metabolism	1.71
VDAG_JR2_Chr8g02840a	Chitin synthase	Polysaccharide metabolism	1.19
VDAG_JR2_Chr8g07660a	Alpha-galactosidase (EC 3.2.1.22) (Melibiase)	Polysaccharide metabolism	1.15
VDAG_JR2_Chr8g09390a	Beta-xylanase (EC 3.2.1.8)	Polysaccharide metabolism	4.22
VDAG_JR2_Chr8g10500a	Glucanase (EC 3.2.1.-)	Polysaccharide metabolism	1.07
VDAG_JR2_Chr1g01080a	Maltose permease	Proton driven symporter	2.77
VDAG_JR2_Chr1g05760a	Galactose-proton symporter	Proton driven symporter	1.70
VDAG_JR2_Chr1g12250a	Choline transport protein	Proton driven symporter	1.50
VDAG_JR2_Chr2g08880a	Maltose permease MAL31	Proton driven symporter	1.34
VDAG_JR2_Chr2g12320a	General alpha-glucoside permease	Proton driven symporter	1.43
VDAG_JR2_Chr3g01050a	Membrane transporter D1	Proton driven symporter	1.34
VDAG_JR2_Chr3g10590a	General alpha-glucoside permease	Proton driven symporter	1.66

**Gene identifier**	**Protein name**	**FunCat description**	**log_2(fold change)**
VDAG_JR2_Chr3g12560a	Maltose permease MAL31	Proton driven symporter	1.21
VDAG_JR2_Chr3g12900a	Alpha-glucosides permease MPH2/3	Proton driven symporter	1.08
VDAG_JR2_Chr4g06500a	Proton myo-inositol cotransporter	Proton driven symporter	1.73
VDAG_JR2_Chr5g03240a	Maltose permease MAL31	Proton driven symporter	1.20
VDAG_JR2_Chr6g08800a	General alpha-glucoside permease	Proton driven symporter	3.19
VDAG_JR2_Chr7g07610a	Choline transport protein	Proton driven symporter	1.12
VDAG_JR2_Chr7g10440a	Sodium/nucleoside cotransporter	Proton driven symporter	1.21
VDAG_JR2_Chr8g01390a	Maltose permease MAL31	Proton driven symporter	3.28
VDAG_JR2_Chr8g07670a	Maltose permease MAL31	Proton driven symporter	1.64
VDAG_JR2_Chr1g00310a	ATP-binding cassette sub-family G member 2	Secondary metabolism	1.11
VDAG_JR2_Chr1g01080a	Maltose permease	Secondary metabolism	2.77
VDAG_JR2_Chr1g01185a	Hexose transporter	Secondary metabolism	1.51
VDAG_JR2_Chr1g01440a	Sugar transporter	Secondary metabolism	1.62
VDAG_JR2_Chr1g01910a	Putative uncharacterized protein	Secondary metabolism	2.28
VDAG_JR2_Chr1g03120a	SERAC1 protein	Secondary metabolism	1.32
VDAG_JR2_Chr1g04380a	Putative uncharacterized protein	Secondary metabolism	1.03
VDAG_JR2_Chr1g05030a	Ferric-chelate reductase	Secondary metabolism	1.93
VDAG_JR2_Chr1g05330a	Cytochrome P450	Secondary metabolism	1.50
VDAG_JR2_Chr1g05350a	Putative uncharacterized protein	Secondary metabolism	2.84
VDAG_JR2_Chr1g05360a	4-coumarate-CoA ligase	Secondary metabolism	1.52
VDAG_JR2_Chr1g05390a	Aldehyde dehydrogenase	Secondary metabolism	4.23
VDAG_JR2_Chr1g05400a	Ankyrin repeat protein	Secondary metabolism	5.15
VDAG_JR2_Chr1g05420a	Putative uncharacterized protein	Secondary metabolism	8.43
VDAG_JR2_Chr1g05480a	Putative uncharacterized protein	Secondary metabolism	1.92
VDAG_JR2_Chr1g05500a	3-oxoacyl-[acyl-carrier-protein] reductase	Secondary metabolism	1.17
VDAG_JR2_Chr1g06490a	L-amino acid oxidase	Secondary metabolism	2.38
VDAG_JR2_Chr1g08670a	Putative uncharacterized protein	Secondary metabolism	1.12
VDAG_JR2_Chr1g08876a	FAD binding domain-containing protein	Secondary metabolism	1.12

Gene identifier	Protein name	FunCat description	log_2(fold change)
VDAG_JR2_Chr1g11700a	Amino acid transporter	Secondary metabolism	1.11
VDAG_JR2_Chr1g12250a	Choline transport protein	Secondary metabolism	1.50
VDAG_JR2_Chr1g13480a	Translation initiation factor eIF-2B subunit family protein	Secondary metabolism	1.54
VDAG_JR2_Chr1g14080a	Putative uncharacterized protein	Secondary metabolism	2.22
VDAG_JR2_Chr1g14630a	C6 zinc finger domain-containing protein	Secondary metabolism	2.29
VDAG_JR2_Chr1g15480a	Putative uncharacterized protein	Secondary metabolism	1.40
VDAG_JR2_Chr1g15850a	ABC transporter CDR4	Secondary metabolism	1.22
VDAG_JR2_Chr1g15970a	Amine oxidase	Secondary metabolism	1.07
VDAG_JR2_Chr1g16560a	Putative uncharacterized protein	Secondary metabolism	1.22
VDAG_JR2_Chr1g17030a	Transcriptional activator protein acu-15	Secondary metabolism	1.94
VDAG_JR2_Chr1g18230a	Glucoamylase (EC 3.2.1.3) (1,4-alpha-D-glucan glucohydrolase) (Glucan 1,4-alpha-glucosidase)	Secondary metabolism	1.40
VDAG_JR2_Chr1g18480a	C6 zinc finger domain-containing protein	Secondary metabolism	1.89
VDAG_JR2_Chr1g18490a	Putative uncharacterized protein	Secondary metabolism	1.69
VDAG_JR2_Chr1g19870a	STB5 protein	Secondary metabolism	1.84
VDAG_JR2_Chr1g21550a	Methyltransferase	Secondary metabolism	1.04
VDAG_JR2_Chr1g22050a	Sugar transporter STL1	Secondary metabolism	2.06
VDAG_JR2_Chr1g22840a	Putative uncharacterized protein	Secondary metabolism	1.56
VDAG_JR2_Chr1g23310a	Putative uncharacterized protein	Secondary metabolism	1.30
VDAG_JR2_Chr1g23860a	Putative uncharacterized protein	Secondary metabolism	2.55
VDAG_JR2_Chr1g23870a	Integral membrane protein	Secondary metabolism	2.52
VDAG_JR2_Chr1g24120a	MAS1 protein	Secondary metabolism	1.10
VDAG_JR2_Chr1g24520a	Sugar transporter	Secondary metabolism	1.70
VDAG_JR2_Chr1g24600a	4-hydroxyacetophenone monooxygenase	Secondary metabolism	3.30
VDAG_JR2_Chr1g24800a	Ent-kaurene oxidase	Secondary metabolism	1.37
VDAG_JR2_Chr1g25900a	Amino acid transporter	Secondary metabolism	1.42
VDAG_JR2_Chr1g26000a	Putative uncharacterized protein	Secondary metabolism	1.35

Gene identifier	Protein name	FunCat description	log_2(fold change)
VDAG_JR2_Chr1g26520a	Cutinase transcription factor 1 alpha	Secondary metabolism	1.74
VDAG_JR2_Chr1g26890a	Pleiotropic ABC multiple drug efflux transporter	Secondary metabolism	1.07
VDAG_JR2_Chr1g27940a	Polyamine oxidase	Secondary metabolism	1.31
VDAG_JR2_Chr1g28170a	Putative uncharacterized protein	Secondary metabolism	1.50
VDAG_JR2_Chr1g28200a	Putative uncharacterized protein	Secondary metabolism	1.29
VDAG_JR2_Chr1g28970a	Alpha-galactosidase (EC 3.2.1.22) (Melibiase)	Secondary metabolism	2.20
VDAG_JR2_Chr1g29400a	NACHT and WD domain-containing protein	Secondary metabolism	1.58
VDAG_JR2_Chr2g02210a	Fungal specific transcription factor domain-containing protein	Secondary metabolism	1.35
VDAG_JR2_Chr2g02540a	Pantothenate transporter liz1	Secondary metabolism	7.40
VDAG_JR2_Chr2g04160a	4-coumarate-CoA ligase	Secondary metabolism	2.58
VDAG_JR2_Chr2g05230a	Putative uncharacterized protein	Secondary metabolism	1.92
VDAG_JR2_Chr2g05870a	Glucoamylase (EC 3.2.1.3) (1,4-alpha-D-glucan glucohydrolase) (Glucan 1,4-alpha-glucosidase)	Secondary metabolism	1.49
VDAG_JR2_Chr2g06830a	Xylosidase/arabinosidase	Secondary metabolism	1.04
VDAG_JR2_Chr2g07210a	DUF341 domain-containing protein	Secondary metabolism	1.61
VDAG_JR2_Chr2g09270a	Putative uncharacterized protein	Secondary metabolism	1.25
VDAG_JR2_Chr2g10500a	Methyltransferase	Secondary metabolism	1.30
VDAG_JR2_Chr2g11750a	UMTA protein	Secondary metabolism	2.00
VDAG_JR2_Chr2g12010a	Trichothecene efflux pump	Secondary metabolism	3.01
VDAG_JR2_Chr2g12320a	General alpha-glucoside permease	Secondary metabolism	1.43
VDAG_JR2_Chr2g12790a	FAD binding domain-containing protein	Secondary metabolism	2.69
VDAG_JR2_Chr2g12840a	Homoprotocatechuate catabolism bifunctional isomerase/decarboxylase	Secondary metabolism	1.51
VDAG_JR2_Chr2g13170a	Putative uncharacterized protein	Secondary metabolism	1.08

Gene identifier	Protein name	FunCat description	$\log_2$(fold change)
VDAG_JR2_Chr2g13190a	Voltage-gated potassium channel subunit beta-3	Secondary metabolism	1.20
VDAG_JR2_Chr2g13240a	Choline dehydrogenase	Secondary metabolism	4.55
VDAG_JR2_Chr3g01490a	Cytochrome P450 52A11	Secondary metabolism	1.27
VDAG_JR2_Chr3g02480a	IBR domain-containing protein	Secondary metabolism	1.56
VDAG_JR2_Chr3g03230a	4-coumarate-CoA ligase	Secondary metabolism	1.49
VDAG_JR2_Chr3g05030a	Phenol 2-monooxygenase	Secondary metabolism	1.58
VDAG_JR2_Chr3g05040a	Cytochrome P450 71B28	Secondary metabolism	1.47
VDAG_JR2_Chr3g06490a	C6 zinc finger domain-containing protein	Secondary metabolism	1.89
VDAG_JR2_Chr3g09580a	Putative uncharacterized protein	Secondary metabolism	1.29
VDAG_JR2_Chr3g10430a	Lactose permease	Secondary metabolism	4.55
VDAG_JR2_Chr3g10460a	Putative uncharacterized protein	Secondary metabolism	1.74
VDAG_JR2_Chr3g11570a	Protein kinase domain-containing protein	Secondary metabolism	2.02
VDAG_JR2_Chr3g11590a	Monooxygenase	Secondary metabolism	5.17
VDAG_JR2_Chr3g11930a	Putative uncharacterized protein	Secondary metabolism	1.04
VDAG_JR2_Chr3g12360a	Cellobiose dehydrogenase	Secondary metabolism	2.27
VDAG_JR2_Chr3g12800a	Putative uncharacterized protein	Secondary metabolism	1.71
VDAG_JR2_Chr3g12810a	FAD binding domain-containing protein	Secondary metabolism	1.55
VDAG_JR2_Chr3g12900a	Alpha-glucosides permease MPH2/3	Secondary metabolism	1.08
VDAG_JR2_Chr3g13250a	L-ornithine 5-monooxygenase	Secondary metabolism	1.64
VDAG_JR2_Chr3g13290a	Diacylglycerol pyrophosphate phosphatase	Secondary metabolism	1.17
VDAG_JR2_Chr4g00210a	Bilirubin oxidase	Secondary metabolism	6.21
VDAG_JR2_Chr4g00490a	Bifunctional P-450:NADPH-P450 reductase	Secondary metabolism	2.42
VDAG_JR2_Chr4g00820a	Putative uncharacterized protein	Secondary metabolism	1.31
VDAG_JR2_Chr4g00850a	Putative uncharacterized protein	Secondary metabolism	2.49
VDAG_JR2_Chr4g01210a	Acetamidase	Secondary metabolism	1.40
VDAG_JR2_Chr4g01490a	Putative uncharacterized protein	Secondary metabolism	1.19

Gene identifier	Protein name	FunCat description	log_2(fold change)
VDAG_JR2_Chr4g01630a	Aerobactin siderophore biosynthesis protein iucB	Secondary metabolism	3.37
VDAG_JR2_Chr4g01750a	Sugar transporter	Secondary metabolism	1.33
VDAG_JR2_Chr4g02590a	Cytochrome P450 52A11	Secondary metabolism	1.46
VDAG_JR2_Chr4g03030a	Bifunctional P-450:NADPH-P450 reductase	Secondary metabolism	1.69
VDAG_JR2_Chr4g04410a	Putative uncharacterized protein	Secondary metabolism	1.70
VDAG_JR2_Chr4g04640a	Aromatic and neutral aliphatic amino acid permease	Secondary metabolism	1.06
VDAG_JR2_Chr4g04720a	Proline-specific permease	Secondary metabolism	1.18
VDAG_JR2_Chr4g04880a	Putative uncharacterized protein	Secondary metabolism	1.44
VDAG_JR2_Chr4g05020a	Lactose regulatory protein LAC9	Secondary metabolism	1.25
VDAG_JR2_Chr4g05150a	Putative uncharacterized protein	Secondary metabolism	1.10
VDAG_JR2_Chr4g05480a	Lipase/esterase	Secondary metabolism	4.16
VDAG_JR2_Chr4g05940a	Alpha-glucosides permease MPH2/3	Secondary metabolism	4.31
VDAG_JR2_Chr4g07460a	FAD binding domain-containing protein	Secondary metabolism	1.27
VDAG_JR2_Chr4g10580a	Putative uncharacterized protein	Secondary metabolism	1.85
VDAG_JR2_Chr4g10620a	Salicylate hydroxylase	Secondary metabolism	3.47
VDAG_JR2_Chr4g10700a	Pisatin demethylase	Secondary metabolism	2.48
VDAG_JR2_Chr4g10730a	Stress responsive A/B barrel domain-containing protein	Secondary metabolism	2.96
VDAG_JR2_Chr4g10760a	Choline transport protein	Secondary metabolism	4.55
VDAG_JR2_Chr4g10770a	Amine oxidase	Secondary metabolism	3.27
VDAG_JR2_Chr4g10780a	Cytochrome P450 76A2	Secondary metabolism	4.85
VDAG_JR2_Chr4g10900a	Vitamin H transporter	Secondary metabolism	3.04
VDAG_JR2_Chr4g10990a	Sulphydryl oxidase	Secondary metabolism	1.48
VDAG_JR2_Chr4g11460a	Amino acid transporter	Secondary metabolism	1.47
VDAG_JR2_Chr4g11570a	Metalloreductase	Secondary metabolism	1.15
VDAG_JR2_Chr4g11940a	Alpha/beta hydrolase	Secondary metabolism	1.51

Gene identifier	Protein name	FunCat description	log_2(fold change)
VDAG_JR2_Chr4g12400a	Oxidoreductase	Secondary metabolism	3.07
VDAG_JR2_Chr5g00030a	Putative uncharacterized protein	Secondary metabolism	1.13
VDAG_JR2_Chr5g00050a	Salicylate hydroxylase	Secondary metabolism	2.02
VDAG_JR2_Chr5g00060a	Aldehyde dehydrogenase	Secondary metabolism	3.15
VDAG_JR2_Chr5g00190a	Glucose transporter	Secondary metabolism	1.09
VDAG_JR2_Chr5g00860a	Glucose oxidase	Secondary metabolism	1.39
VDAG_JR2_Chr5g00970a	Choline monooxygenase	Secondary metabolism	1.93
VDAG_JR2_Chr5g01240a	Benomyl/methotrexate resistance protein	Secondary metabolism	2.29
VDAG_JR2_Chr5g01310a	C6 zinc finger protein	Secondary metabolism	1.10
VDAG_JR2_Chr5g03740a	Lactose permease	Secondary metabolism	1.89
VDAG_JR2_Chr5g04160a	Proton myo-inositol cotransporter	Secondary metabolism	2.51
VDAG_JR2_Chr5g04590a	Mannan endo-1,6-alpha-mannosidase DCW1	Secondary metabolism	1.77
VDAG_JR2_Chr5g05090a	Putative uncharacterized protein	Secondary metabolism	1.61
VDAG_JR2_Chr5g05100a	Quinate permease	Secondary metabolism	1.56
VDAG_JR2_Chr5g06930a	Choline transport protein	Secondary metabolism	4.08
VDAG_JR2_Chr5g06940a	Monoamine oxidase N	Secondary metabolism	4.95
VDAG_JR2_Chr5g08280a	Zinc finger transcription factor 1	Secondary metabolism	1.35
VDAG_JR2_Chr5g10570a	Ankyrin repeat protein	Secondary metabolism	1.94
VDAG_JR2_Chr5g11040a	Alcohol oxidase	Secondary metabolism	3.50
VDAG_JR2_Chr5g11880a	Choline dehydrogenase	Secondary metabolism	1.24
VDAG_JR2_Chr6g01130a	FAD binding domain-containing protein	Secondary metabolism	5.17
VDAG_JR2_Chr6g01280a	Thymine dioxygenase	Secondary metabolism	1.95
VDAG_JR2_Chr6g01290a	Lactose permease	Secondary metabolism	1.17
VDAG_JR2_Chr6g01850a	Putative uncharacterized protein	Secondary metabolism	1.22
VDAG_JR2_Chr6g02300a	Choline dehydrogenase	Secondary metabolism	2.31
VDAG_JR2_Chr6g04210a	Siderophore iron transporter mirB	Secondary metabolism	1.70
VDAG_JR2_Chr6g05120a	Putative uncharacterized protein	Secondary metabolism	1.38
VDAG_JR2_Chr6g05300a	Putative uncharacterized protein	Secondary metabolism	1.80

Gene identifier	Protein name	FunCat description	log_2(fold change)
VDAG_JR2_Chr6g05470a	Short chain dehydrogenase	Secondary metabolism	1.20
VDAG_JR2_Chr6g05780a	Cyclopentanone 1,2-monooxygenase	Secondary metabolism	2.13
VDAG_JR2_Chr6g06030a	Averantin oxidoreductase	Secondary metabolism	1.57
VDAG_JR2_Chr6g06060a	4-trimethylaminobutyraldehyde dehydrogenase	Secondary metabolism	1.39
VDAG_JR2_Chr6g08410a	Putative uncharacterized protein	Secondary metabolism	2.00
VDAG_JR2_Chr6g08420a	Amine oxidase B	Secondary metabolism	1.26
VDAG_JR2_Chr6g08610a	Phenylacetone monooxygenase	Secondary metabolism	1.13
VDAG_JR2_Chr6g08800a	General alpha-glucoside permease	Secondary metabolism	3.19
VDAG_JR2_Chr6g09100a	Putative uncharacterized protein	Secondary metabolism	1.69
VDAG_JR2_Chr6g09120a	Aromatic and neutral aliphatic amino acid permease	Secondary metabolism	1.11
VDAG_JR2_Chr6g09680a	Alcohol oxidase	Secondary metabolism	1.47
VDAG_JR2_Chr6g09710a	Quinate permease	Secondary metabolism	1.04
VDAG_JR2_Chr6g10160a	Acetylesterase	Secondary metabolism	1.79
VDAG_JR2_Chr6g10890a	Putative uncharacterized protein	Secondary metabolism	2.46
VDAG_JR2_Chr7g00700a	Putative uncharacterized protein	Secondary metabolism	1.18
VDAG_JR2_Chr7g01150a	Putative uncharacterized protein	Secondary metabolism	1.20
VDAG_JR2_Chr7g02910a	Putative uncharacterized protein	Secondary metabolism	1.07
VDAG_JR2_Chr7g03180a	FMP42 protein	Secondary metabolism	4.22
VDAG_JR2_Chr7g03200a	Putative uncharacterized protein	Secondary metabolism	2.41
VDAG_JR2_Chr7g03570a	Cellobiose dehydrogenase	Secondary metabolism	1.03
VDAG_JR2_Chr7g03610a	Riboflavin transporter MCH5	Secondary metabolism	2.36
VDAG_JR2_Chr7g05220a	TOL protein	Secondary metabolism	1.41
VDAG_JR2_Chr7g05440a	Beta,beta-carotene 9',10'-dioxygenase	Secondary metabolism	1.42
VDAG_JR2_Chr7g05450a	Averantin oxidoreductase	Secondary metabolism	1.72
VDAG_JR2_Chr7g07610a	Choline transport protein	Secondary metabolism	1.12
VDAG_JR2_Chr7g08060a	Putative uncharacterized protein	Secondary metabolism	2.03
VDAG_JR2_Chr7g09570a	Sugar transporter	Secondary metabolism	1.01

Gene identifier	Protein name	FunCat description	$\log_2$(fold change)
VDAG_JR2_Chr7g10250a	Putative uncharacterized protein	Secondary metabolism	1.63
VDAG_JR2_Chr8g00630a	Fluconazole resistance protein	Secondary metabolism	3.15
VDAG_JR2_Chr8g00880a	Lactose permease	Secondary metabolism	1.88
VDAG_JR2_Chr8g00990a	Sugar transporter	Secondary metabolism	2.49
VDAG_JR2_Chr8g01050a	Phthalate transporter	Secondary metabolism	1.36
VDAG_JR2_Chr8g01500a	Putative uncharacterized protein	Secondary metabolism	1.48
VDAG_JR2_Chr8g01610a	Maleylacetate reductase	Secondary metabolism	2.27
VDAG_JR2_Chr8g01660a	Putative uncharacterized protein	Secondary metabolism	2.93
VDAG_JR2_Chr8g02120a	Aldehyde dehydrogenase	Secondary metabolism	1.11
VDAG_JR2_Chr8g02280a	Vitamin H transporter 1	Secondary metabolism	1.44
VDAG_JR2_Chr8g02900a	Putative uncharacterized protein	Secondary metabolism	2.18
VDAG_JR2_Chr8g07670a	Maltose permease MAL31	Secondary metabolism	1.64
VDAG_JR2_Chr8g08860a	Gibberellin 20 oxidase	Secondary metabolism	1.39
VDAG_JR2_Chr8g09050a	Averantin oxidoreductase	Secondary metabolism	7.72
VDAG_JR2_Chr8g09440a	Putative uncharacterized protein	Secondary metabolism	1.48
VDAG_JR2_Chr8g10170a	Siderophore iron transporter mirB	Secondary metabolism	2.25
VDAG_JR2_Chr8g10320a	Putative uncharacterized protein	Secondary metabolism	2.99
VDAG_JR2_Chr8g10350a	Phthalate transporter	Secondary metabolism	3.85
VDAG_JR2_Chr8g11120a	Integral membrane protein	Secondary metabolism	2.51
VDAG_JR2_Chr1g00390a	Iron transport multicopper oxidase FET3	Transport facilities	4.06
VDAG_JR2_Chr1g00400a	Plasma membrane iron permease	Transport facilities	2.03
VDAG_JR2_Chr1g01070a	Putative uncharacterized protein	Transport facilities	4.75
VDAG_JR2_Chr1g01080a	Maltose permease	Transport facilities	2.77
VDAG_JR2_Chr1g01185a	Hexose transporter	Transport facilities	1.51
VDAG_JR2_Chr1g01330a	NADH-cytochrome b5 reductase	Transport facilities	1.13
VDAG_JR2_Chr1g01440a	Sugar transporter	Transport facilities	1.62
VDAG_JR2_Chr1g01710a	Inner membrane transport protein yfaV	Transport facilities	1.52
VDAG_JR2_Chr1g02220a	Sugar transporter	Transport facilities	2.06
VDAG_JR2_Chr1g05010a	Peptide transporter PTR2	Transport facilities	3.23

Gene identifier	Protein name	FunCat description	$\log_2$(fold change)
VDAG_JR2_Chr1g05760a	Galactose-proton symporter	Transport facilities	1.70
VDAG_JR2_Chr1g08890a	Putative uncharacterized protein	Transport facilities	1.29
VDAG_JR2_Chr1g10700a	Solute carrier family 12 member 6	Transport facilities	2.26
VDAG_JR2_Chr1g11690a	Putative uncharacterized protein	Transport facilities	1.34
VDAG_JR2_Chr1g12250a	Choline transport protein	Transport facilities	1.50
VDAG_JR2_Chr1g15370a	Putative uncharacterized protein	Transport facilities	1.59
VDAG_JR2_Chr1g18850a	Putative uncharacterized protein	Transport facilities	1.14
VDAG_JR2_Chr1g22050a	Sugar transporter STL1	Transport facilities	2.06
VDAG_JR2_Chr1g22590a	Vacuolar calcium ion transporter	Transport facilities	3.81
VDAG_JR2_Chr1g23370a	Aquaporin-2	Transport facilities	3.31
VDAG_JR2_Chr1g24520a	Sugar transporter	Transport facilities	1.70
VDAG_JR2_Chr1g24720a	Allantoate permease	Transport facilities	1.03
VDAG_JR2_Chr1g24790a	High-affinity nicotinic acid transporter	Transport facilities	2.54
VDAG_JR2_Chr1g26920a	High-affinity glucose transporter SNF3	Transport facilities	1.27
VDAG_JR2_Chr2g02540a	Pantothenate transporter liz1	Transport facilities	7.40
VDAG_JR2_Chr2g02970a	Integral membrane protein	Transport facilities	1.18
VDAG_JR2_Chr2g03860a	Inner membrane transport protein yfaV	Transport facilities	2.02
VDAG_JR2_Chr2g04380a	Integral membrane protein	Transport facilities	1.88
VDAG_JR2_Chr2g04730a	Zinc/iron transporter protein	Transport facilities	1.13
VDAG_JR2_Chr2g08730a	Putative uncharacterized protein	Transport facilities	1.43
VDAG_JR2_Chr2g08880a	Maltose permease MAL31	Transport facilities	1.34
VDAG_JR2_Chr2g09810a	Inner membrane protein yicO	Transport facilities	1.44
VDAG_JR2_Chr2g12320a	General alpha-glucoside permease	Transport facilities	1.43
VDAG_JR2_Chr2g13170a	Putative uncharacterized protein	Transport facilities	1.08
VDAG_JR2_Chr3g01050a	Membrane transporter D1	Transport facilities	1.34
VDAG_JR2_Chr3g05190a	Sodium/calcium exchanger protein	Transport facilities	1.17
VDAG_JR2_Chr3g10590a	General alpha-glucoside permease	Transport facilities	1.66
VDAG_JR2_Chr3g12560a	Maltose permease MAL31	Transport facilities	1.21
VDAG_JR2_Chr3g12800a	Putative uncharacterized protein	Transport facilities	1.71

Gene identifier	Protein name	FunCat description	log_2(fold change)
VDAG_JR2_Chr3g12900a	Alpha-glucosides permease MPH2/3	Transport facilities	1.08
VDAG_JR2_Chr4g00720a	Phosphate transporter family protein	Transport facilities	1.67
VDAG_JR2_Chr4g01150a	Glucose/galactose transporter	Transport facilities	1.91
VDAG_JR2_Chr4g01620a	Sulfate permease	Transport facilities	3.70
VDAG_JR2_Chr4g01750a	Sugar transporter	Transport facilities	1.33
VDAG_JR2_Chr4g01780a	Aquaporin-1	Transport facilities	1.14
VDAG_JR2_Chr4g02440a	Putative uncharacterized protein	Transport facilities	1.34
VDAG_JR2_Chr4g03140a	Carnitine O-palmitoyltransferase I	Transport facilities	1.14
VDAG_JR2_Chr4g05930a	Putative uncharacterized protein	Transport facilities	3.10
VDAG_JR2_Chr4g05940a	Alpha-glucosides permease MPH2/3	Transport facilities	4.31
VDAG_JR2_Chr4g06500a	Proton myo-inositol cotransporter	Transport facilities	1.73
VDAG_JR2_Chr4g07840a	Potassium transporter 1	Transport facilities	1.47
VDAG_JR2_Chr4g10760a	Choline transport protein	Transport facilities	4.55
VDAG_JR2_Chr4g10900a	Vitamin H transporter	Transport facilities	3.04
VDAG_JR2_Chr4g11690a	Plasma membrane iron permease	Transport facilities	1.05
VDAG_JR2_Chr4g11820a	General amino-acid permease GAP1	Transport facilities	2.99
VDAG_JR2_Chr4g11930a	Integral membrane protein	Transport facilities	1.68
VDAG_JR2_Chr4g12110a	High-affinity nicotinic acid transporter	Transport facilities	1.50
VDAG_JR2_Chr4g12160a	Ammonium transporter 1	Transport facilities	2.22
VDAG_JR2_Chr4g12390a	Putative uncharacterized protein	Transport facilities	8.31
VDAG_JR2_Chr5g00190a	Glucose transporter	Transport facilities	1.09
VDAG_JR2_Chr5g01240a	Benomyl/methotrexate resistance protein	Transport facilities	2.29
VDAG_JR2_Chr5g01900a	High-affinity potassium transport protein	Transport facilities	3.47
VDAG_JR2_Chr5g01910a	Na+/H+ antiporter	Transport facilities	2.53
VDAG_JR2_Chr5g03240a	Maltose permease MAL31	Transport facilities	1.20
VDAG_JR2_Chr5g03740a	Lactose permease	Transport facilities	1.89
VDAG_JR2_Chr5g04160a	Proton myo-inositol cotransporter	Transport facilities	2.51
VDAG_JR2_Chr5g05790a	Membrane transporter	Transport facilities	1.24
VDAG_JR2_Chr5g06010a	DUF250 domain membrane protein	Transport facilities	1.31

Gene identifier	Protein name	FunCat description	$\log_2$(fold change)
VDAG_JR2_Chr5g06930a	Choline transport protein	Transport facilities	4.08
VDAG_JR2_Chr5g07790a	Solute carrier family 35 member E3	Transport facilities	1.61
VDAG_JR2_Chr5g11480a	Peroxisomal-coenzyme A synthetase	Transport facilities	2.36
VDAG_JR2_Chr5g12230a	Na+/H+ antiporter	Transport facilities	1.49
VDAG_JR2_Chr6g01260a	Pantothenate transporter liz1	Transport facilities	2.12
VDAG_JR2_Chr6g01290a	Lactose permease	Transport facilities	1.17
VDAG_JR2_Chr6g02110a	Thiamine transporter	Transport facilities	1.23
VDAG_JR2_Chr6g02450a	Putative uncharacterized protein	Transport facilities	1.17
VDAG_JR2_Chr6g03600a	Low-affinity potassium transport protein	Transport facilities	1.13
VDAG_JR2_Chr6g04250a	Pantothenate transporter liz1	Transport facilities	3.63
VDAG_JR2_Chr6g08800a	General alpha-glucoside permease	Transport facilities	3.19
VDAG_JR2_Chr6g09100a	Putative uncharacterized protein	Transport facilities	1.69
VDAG_JR2_Chr6g09380a	Allantoate permease	Transport facilities	1.29
VDAG_JR2_Chr6g09800a	K+/H+ antiporter 1	Transport facilities	2.20
VDAG_JR2_Chr6g10740a	High-affinity glucose transporter RGT2	Transport facilities	6.81
VDAG_JR2_Chr7g00030a	Pantothenate transporter liz1	Transport facilities	1.02
VDAG_JR2_Chr7g02360a	Oligopeptide transporter 2	Transport facilities	1.59
VDAG_JR2_Chr7g02750a	Low-affinity potassium transport protein	Transport facilities	1.50
VDAG_JR2_Chr7g03220a	Allantoate permease	Transport facilities	1.78
VDAG_JR2_Chr7g03610a	Riboflavin transporter MCH5	Transport facilities	2.36
VDAG_JR2_Chr7g07610a	Choline transport protein	Transport facilities	1.12
VDAG_JR2_Chr7g09570a	Sugar transporter	Transport facilities	1.01
VDAG_JR2_Chr7g10310a	Amino-acid permease inda1	Transport facilities	1.13
VDAG_JR2_Chr8g00990a	Sugar transporter	Transport facilities	2.49
VDAG_JR2_Chr8g01050a	Phthalate transporter	Transport facilities	1.36
VDAG_JR2_Chr8g01260a	L-fucose transporter	Transport facilities	5.47
VDAG_JR2_Chr8g01390a	Maltose permease MAL31	Transport facilities	3.28
VDAG_JR2_Chr8g01840a	Sugar transporter family protein	Transport facilities	1.33
VDAG_JR2_Chr8g02280a	Vitamin H transporter 1	Transport facilities	1.44

Gene identifier	Protein name	FunCat description	log_2(fold change)
VDAG_JR2_Chr8g02900a	Putative uncharacterized protein	Transport facilities	2.18
VDAG_JR2_Chr8g06660a	Ammonium transporter MEP1	Transport facilities	1.29
VDAG_JR2_Chr8g07670a	Maltose permease MAL31	Transport facilities	1.64
VDAG_JR2_Chr8g09210a	Carboxylic acid transport protein	Transport facilities	7.57
VDAG_JR2_Chr8g10350a	Phthalate transporter	Transport facilities	3.85
VDAG_JR2_Chr8g11110a	Allantoate permease	Transport facilities	1.96
VDAG_JR2_Chr8g11120a	Integral membrane protein	Transport facilities	2.51
VDAG_JR2_Chr1g00680a	CHIP6 protein	Virulence, disease factors	1.16
VDAG_JR2_Chr1g02740a	Urease (EC 3.5.1.5) (Urea amidohydrolase)	Virulence, disease factors	1.25
VDAG_JR2_Chr1g11690a	Putative uncharacterized protein	Virulence, disease factors	1.34
VDAG_JR2_Chr1g18680a	PBSP domain-containing protein	Virulence, disease factors	1.51
VDAG_JR2_Chr1g21680a	UDP-glucose,sterol transferase	Virulence, disease factors	1.95
VDAG_JR2_Chr1g22490a	Acetylxylan esterase	Virulence, disease factors	1.39
VDAG_JR2_Chr1g22650a	D-arabinono-1,4-lactone oxidase	Virulence, disease factors	1.84
VDAG_JR2_Chr1g23870a	Integral membrane protein	Virulence, disease factors	2.52
VDAG_JR2_Chr1g27530a	Putative uncharacterized protein	Virulence, disease factors	3.03
VDAG_JR2_Chr1g28060a	Glucan 1,3-beta-glucosidase	Virulence, disease factors	2.38
VDAG_JR2_Chr1g29070a	Putative uncharacterized protein	Virulence, disease factors	2.11
VDAG_JR2_Chr1g29680a	Glucan 1,3-beta-glucosidase	Virulence, disease factors	1.95
VDAG_JR2_Chr2g02970a	Integral membrane protein	Virulence, disease factors	1.18
VDAG_JR2_Chr2g04380a	Integral membrane protein	Virulence, disease factors	1.88
VDAG_JR2_Chr2g08750a	FAD/FMN-containing dehydrogenase	Virulence, disease factors	2.33
VDAG_JR2_Chr2g12010a	Trichothecene efflux pump	Virulence, disease factors	3.01
VDAG_JR2_Chr3g06090a	UDP-glucose,sterol transferase	Virulence, disease factors	2.94
VDAG_JR2_Chr3g06110a	Cyanide hydratase	Virulence, disease factors	1.55
VDAG_JR2_Chr3g12800a	Putative uncharacterized protein	Virulence, disease factors	1.71
VDAG_JR2_Chr4g02440a	Putative uncharacterized protein	Virulence, disease factors	1.34
VDAG_JR2_Chr4g03780a	CHIP6 protein	Virulence, disease factors	1.10

Gene identifier	Protein name	FunCat description	log_2(fold change)
VDAG_JR2_Chr4g05930a	Putative uncharacterized protein	Virulence, disease factors	3.10
VDAG_JR2_Chr4g09730a	Putative uncharacterized protein	Virulence, disease factors	1.24
VDAG_JR2_Chr4g11930a	Integral membrane protein	Virulence, disease factors	1.68
VDAG_JR2_Chr5g02420a	UDP-glucose,sterol transferase	Virulence, disease factors	1.90
VDAG_JR2_Chr5g07180a	Rhamnogalacturonan acetylesterase	Virulence, disease factors	1.63
VDAG_JR2_Chr6g00450a	Glucan 1,3-beta-glucosidase	Virulence, disease factors	1.83
VDAG_JR2_Chr6g03100a	Putative uncharacterized protein	Virulence, disease factors	3.08
VDAG_JR2_Chr7g01150a	Putative uncharacterized protein	Virulence, disease factors	1.20
VDAG_JR2_Chr7g01470a	Integral membrane protein	Virulence, disease factors	1.30
VDAG_JR2_Chr8g01660a	Putative uncharacterized protein	Virulence, disease factors	2.93
VDAG_JR2_Chr8g10310a	Fatty acid synthase S-acetyltransferase	Virulence, disease factors	2.70
VDAG_JR2_Chr8g11120a	Integral membrane protein	Virulence, disease factors	2.51

Candidates mentioned in the article are highlighted in light blue.

Table S2: List of *V. dahliae* genes strongly induced in tomato xylem sap with domains of derived proteins and their putative function.

Gene identifier	Putative functional category	Domains	log_2(fold change)	Signal peptide
**Hydrophobin**				
VDAG_JR2_Chr1g24730a/ VDH5	Hydrophobin	Dense granule Gra6 protein (PR01746, IPR008119), Cerato-ulmin hydrophobin family (PF06766, IPR010636)	5.99	yes
VDAG_JR2_Chr2g02500a/ VDH1	Hydrophobin	Cerato-ulmin hydrophobin family (PF06766, IPR010636)	5.81	yes
VDAG_JR2_Chr3g09420a/ VDH4	Hydrophobin	Cerato-ulmin hydrophobin family (PF06766, IPR010636)	6.85	no, but extracellular prediction
VDAG_JR2_Chr4g07990a/ VDH2	Hydrophobin	Cerato-ulmin hydrophobin family (PF06766, IPR010636)	4.54	yes
**Metabolism**				
VDAG_JR2_Chr1g06240a	Metabolism (carbohydrate)	Glycoside hydrolase, family 3, N-terminal (PF00933, IPR001764), Glycoside hydrolase family 3 C-terminal domain (PF01915, IPR002772), Fibronectin type III-like domain (PF14310, IPR026891)	4.43	no
VDAG_JR2_Chr1g29470a	Metabolism (purine)	Guanylate kinase/L-type calcium channel beta subunit (PF00625, IPR008145)	4.56	no
VDAG_JR2_Chr3g12130a	Metabolism (RNA)	Guanine-specific ribonuclease N1/T1/U2 (PF00545, IPR000026)	4.36	yes
VDAG_JR2_Chr5g09380a	Metabolism (carbohydrate)	Glycoside hydrolase, family 61 (PF03443, IPR005103)	4.18	no, but extracellular prediction
VDAG_JR2_Chr6g01140a	Metabolism (aromatic compound)/Redox process	Extradiol ring-cleavage dioxygenase, class III enzyme, subunit B (PF02900, IPR004183)	5.01	no

Gene identifier	Putative functional category	Domains	log_2(fold change)	Signal peptide
VDAG_JR2_Chr6g06580a	Metabolism (phospholipid)	Phospholipase A2, prokaryotic/fungal (PF09056, IPR015141)	4.20	yes
VDAG_JR2_Chr7g00075a	Metabolism (carbohydrate)	Glycoside hydrolase, family 3, N-terminal (PF00933, IPR001764)	5.09	no, but extracellular prediction
VDAG_JR2_Chr7g01620a	Metabolism (carbohydrate)	Alginate lyase domain (PF05426, IPR008397)	7.04	yes
VDAG_JR2_Chr8g09390a	Metabolism (carbohydrate)	Glycoside hydrolase family 10 domain (PF00331, IPR001000), Cellulose-binding domain, fungal (PF00734, IPR000254)	4.22	yes
VDAG_JR2_Chr8g09870a	Metabolism (amino acid)	Histidinol dehydrogenase (PF00815, IPR012131)	4.63	no
**Others**				
VDAG_JR2_Chr1g23760a	Others (iron storage and detoxification)	Ferritin-like (PF13668, IPR012347)	5.93	yes
VDAG_JR2_Chr2g04040a	Others (adhesion)	Agglutinin-like protein repeat (PF05792, IPR008440)	8.03	no, but extracellular prediction
VDAG_JR2_Chr3g06480a	Others (stress response)	RTA-like protein (PF04479, IPR007568)	9.46	no
VDAG_JR2_Chr6g01130a	Others (decarboxylation)	FAD-binding domain (PF01494, IPR002938), Acetoacetate decarboxylase (PF06314, IPR010451)	5.17	no
VDAG_JR2_Chr7g01630a	Others (virulence: pectin lyase fold/virulence factor superfamily)	Right-handed beta helix domain (PF13229, IPR039448)	7.27	no, but extracellular prediction
VDAG_JR2_Chr8g09420a	Others (secondary metabolism)	Mycotoxin biosynthesis protein UstYa-like (PF11807, IPR021765)	4.08	no
VDAG_JR2_Chr8g09430a	Others (dephosphorylation)	Alkaline phosphatase (PF00245, IPR001952)	4.83	no

Gene identifier	Putative functional category	Domains	log_2(fold change)	Signal peptide
**Proteolysis**				
VDAG_JR2_Chr1g11650a	Proteolysis	Peptidase M43, pregnancy-associated plasma-A (PF05572, IPR008754)	5.39	yes
VDAG_JR2_Chr1g21900a	Proteolysis	Peptidase M43, pregnancy-associated plasma-A (PF05572, IPR008754)	4.87	yes
VDAG_JR2_Chr4g03040a	Proteolysis	Peptidase S8 propeptide/proteinase inhibitor I9 (PF05922, IPR010259), Peptidase S8/S53 domain (PF00082, IPR000209)	4.14	yes
VDAG_JR2_Chr4g04480a	Proteolysis	Peptidase S8/S53 domain (PF00082, IPR000209)	5.32	no
VDAG_JR2_Chr4g11050a	Proteolysis	Peptidase M14, carboxypeptidase A (PF00246, IPR000834)	5.64	yes
VDAG_JR2_Chr4g11220a	Proteolysis	Peptidase M28 (PF04389, IPR007484)	4.51	yes
VDAG_JR2_Chr5g01660a	Proteolysis	Peptidase M14, carboxypeptidase A (PF00246, IPR000834)	6.60	yes
VDAG_JR2_Chr5g03150a	Proteolysis	Peptidase M43, pregnancy-associated plasma-A (PF05572, IPR008754)	4.27	no, but extracellular prediction
VDAG_JR2_Chr5g09960a	Proteolysis	Peptidase S41 family protein (PTHR37049)	4.21	yes
VDAG_JR2_Chr5g09970a	Proteolysis	Peptidase inhibitor I66 (PF16850, IPR031755)	6.56	no
VDAG_JR2_Chr5g09980a	Proteolysis	Serine proteases, trypsin domain (PF00089, IPR001254)	9.37	yes
**Redox process**				
VDAG_JR2_Chr1g00390a	Redox process/Transport (ion)	Multicopper oxidase, type 1 (PF00394, IPR001117), Multicopper oxidase, type 2 (PF07731, IPR011706), Multicopper oxidase, type 3 (PF07732, IPR011707)	4.06	yes
VDAG_JR2_Chr1g05390a	Redox process	Aldehyde dehydrogenase domain (PF00171, IPR015590)	4.23	no

Gene identifier	Putative functional category	Domains	log_2(fold change)	Signal peptide
VDAG_JR2_Chr1g21690a	Redox process	FMN-dependent dehydrogenase (PF01070, IPR000262)	4.12	yes
VDAG_JR2_Chr2g13240a	Redox process	Glucose-methanol-choline oxidoreductase, N-terminal (PF00732, IPR000172), Glucose-methanol-choline oxidoreductase, C-terminal (PF05199, IPR007867)	4.55	yes
VDAG_JR2_Chr3g11590a	Redox process	Pyridine nucleotide-disulphide oxidoreductase (PF13738)	5.17	no
VDAG_JR2_Chr4g00210a	Redox process	Multicopper oxidase, type 1 (PF00394, IPR001117), Multicopper oxidase, type 2 (PF07731, IPR011706), Multicopper oxidase, type 3 (PF07732, IPR011707)	6.21	yes
VDAG_JR2_Chr4g04660a	Redox process	FMN-dependent dehydrogenase (PF01070, IPR000262)	4.01	yes
VDAG_JR2_Chr4g10780a	Redox process	Cytochrome P450 (PF00067, IPR001128)	4.85	no
VDAG_JR2_Chr5g06940a	Redox process	Amine oxidase (PF01593, IPR002937)	4.95	no
VDAG_JR2_Chr8g09050a	Redox process	Cytochrome P450 (PF00067, IPR001128)	7.72	no
VDAG_JR2_Chr8g10330a	Redox process	6-phosphogluconate dehydrogenase, NADP-binding (PF03446, IPR006115), 3-hydroxyisobutyrate dehydrogenase, NAD-binding domain (PF14833, IPR029154), Four-carbon acid sugar kinase, N-terminal domain (PF07005, IPR010737), Nucleotide-binding C-terminal domain (PF17042, IPR031475)	4.02	no
**Transcription**				
VDAG_JR2_Chr3g06100a	Transcription	Zn(2)-C6 fungal-type DNA-binding domain (PF00172, IPR001138), Fungal transcription factor (PF11951, IPR021858)	4.83	no

Gene identifier	Putative functional category	Domains	log_2(fold change)	Signal peptide
VDAG_JR2_Chr8g09060a	Transcription	Zn(2)-C6 fungal-type DNA-binding domain (PF00172, IPR001138)	4.48	no
**Transport**				
VDAG_JR2_Chr1g01070a	Transport (transmembrane)	Major facilitator superfamily (PF07690, IPR011701)	4.75	no
VDAG_JR2_Chr1g05420a	Transport (transmembrane)	Amino acid transporter, transmembrane domain (PF01490, IPR013057)	8.43	no
VDAG_JR2_Chr1g24150a	Transport (transmembrane)	Purine-cytosine permease (PF02133, IPR001248)	6.18	no
VDAG_JR2_Chr1g24740a	Transport (transmembrane)	Major facilitator superfamily (PF07690, IPR011701)	4.32	no
VDAG_JR2_Chr2g02540a	Transport (transmembrane)	Major facilitator superfamily (PF07690, IPR011701)	7.40	no
VDAG_JR2_Chr2g04500a	Transport (transmembrane)	Major facilitator, sugar transporter-like (PF00083, IPR005828)	4.34	no
VDAG_JR2_Chr3g10430a	Transport (transmembrane)	Major facilitator, sugar transporter-like (PF00083, IPR005828)	4.55	no
VDAG_JR2_Chr4g05940a	Transport (transmembrane)	Major facilitator, sugar transporter-like (PF00083, IPR005828)	4.31	no
VDAG_JR2_Chr4g09800a	Transport (transmembrane)	Major facilitator superfamily (PF07690, IPR011701)	4.30	no
VDAG_JR2_Chr4g10760a	Transport (transmembrane)	Amino acid/polyamine transporter I (PF13520, IPR002293)	4.55	no
VDAG_JR2_Chr4g12390a	Transport (transmembrane)	Major facilitator superfamily (PF07690, IPR011701)	8.31	no
VDAG_JR2_Chr5g03250a	Transport (transmembrane)	MFS transporter superfamily (SSF103473, IPR036259)	4.37	no
VDAG_JR2_Chr5g06930a	Transport (transmembrane)	Amino acid/polyamine transporter I (PF13520, IPR002293)	4.08	no

Gene identifier	Putative functional category	Domains	$\log_2$(fold change)	Signal peptide
VDAG_JR2_Chr6g10740a	Transport (transmembrane)	Major facilitator, sugar transporter-like (PF00083, IPR005828)	6.81	no
VDAG_JR2_Chr7g03180a	Transport (transmembrane)	Major facilitator superfamily (PF07690, IPR011701)	4.22	no
VDAG_JR2_Chr8g01260a	Transport (transmembrane)	Major facilitator superfamily (PF07690, IPR011701)	5.47	no
VDAG_JR2_Chr8g09210a	Transport (transmembrane)	Major facilitator, sugar transporter-like (PF00083, IPR005828)	7.57	no
**Unknown function**				
VDAG_JR2_Chr1g01520a	Unknown function	-	4.35	no
VDAG_JR2_Chr1g04580a	Unknown function	-	4.67	no
VDAG_JR2_Chr1g05400a	Unknown function	NACHT nucleoside triphosphatase (PF05729, IPR007111)	5.15	no
VDAG_JR2_Chr1g11660a	Unknown function (potential membrane protein)	-	4.10	yes
VDAG_JR2_Chr2g03100a	Unknown function (potentially extracellular)	Protein of unknown function DUF4419	6.94	yes
VDAG_JR2_Chr2g04030a	Unknown function	-	6.94	no
VDAG_JR2_Chr2g04050a	Unknown function (potential membrane protein)	-	8.46	yes
VDAG_JR2_Chr2g11700a	Unknown function	-	4.24	no
VDAG_JR2_Chr2g12700a	Unknown function (potential extracellular, small, C-rich protein; no signal peptide)	-	4.75	no, but extracellular prediction
VDAG_JR2_Chr3g05620a	Unknown function (potentially extracellular)	Knottin, scorpion toxin-like superfamily (IPR036574)	7.36	yes
VDAG_JR2_Chr3g12210a	Unknown function (potentially extracellular)	Carboxylesterase, type B (PF00135, IPR002018)	4.43	yes
VDAG_JR2_Chr4g01640a	Unknown function	-	4.68	no

Gene identifier	Putative functional category	Domains	$\log_2$(fold change)	Signal peptide
VDAG_JR2_Chr4g02770a	Unknown function	Domain of unknown function DUF1524 (PF07510, IPR011089)	5.39	no
VDAG_JR2_Chr4g05480a	Unknown function	Alpha/beta hydrolase fold-3 (PF07859, IPR013094)	4.16	no
VDAG_JR2_Chr4g11330a	Unknown function (potential effector)	-	5.34	yes
VDAG_JR2_Chr5g05880a	Unknown function (potentially extracellular)	Family of unknown function DUF5695 (PF18951)	4.33	yes
VDAG_JR2_Chr5g07170a	Unknown function	-	5.68	no
VDAG_JR2_Chr7g01190a	Unknown function (potentially extracellular)	-	4.56	yes
VDAG_JR2_Chr8g01400a	Unknown function	-	5.89	no
VDAG_JR2_Chr8g01770a	Unknown function	Histidine phosphatase superfamily, clade-1 (PF00300, IPR013078)	4.19	no
VDAG_JR2_Chr8g01900a	Unknown function	-	4.24	no
VDAG_JR2_Chr8g09410a	Unknown function (potentially extracellular)	-	5.41	yes
VDAG_JR2_Chr8g09730a	Unknown function	-	4.46	no

Candidates mentioned in the article are highlighted in light blue.

Table S3: Primer oligonucleotides used in this study.

Primer name	Primer sequence (5’ → 3’)	Length (-mer)	Overhang to
IM28	**ATT CTT AAT TAA GAT** GGA GAA AAG GGC AAT GGT	33	pME4564
IM29	**AGA TCC CCG GGT ACC** TTT GAT GGT TGG GGG TTG	33	*^{p}gpdA*
IM30	**AGG TAA TCC TTC TTT** GCG TCG CGG TTC CCG GAC	33	*trpC^{t}*
IM31	**AGG ACT TCT AGA AGG** CAC GTT TCT AAC ACG TGC CGC	36	pME4564
IM36	**ATT CTT AAT TAA GAT** AGC TTG TTT CTG CTG ATC	33	pME4564
IM37	**ACC GGT CAC TGT ACA** GGC CAC CGA CTT TAT ATA	33	*^{p}gpdA*
IM38	**AGG TAA TCC TTC TTT** GTA CCA GCC CGG CTG TGA	33	*trpC^{t}*
IM39	**AGG ACT TCT AGA AGG** ATT GCG AGA CGC TTA GCC	33	pME4564
IM52	ATG AAG TTC ACC ACT GTC GC	20	-
IM53	TTA GGC GGC GAC GCC AAC	18	-
IM54	ATG CCA GGT CTA GAG CTC TC	20	-
IM76	**ATT CTT AAT TAA GAT** CCT CAT CTG TTG AAT TTC GT	35	pME4564
IM77	**ATA TCA GTT AAC GTC** CTT GGG ATA GTA AAT AGA TAC TG	38	*^{p}trpC*
IM78	**ACC AGC CCC TGG GTT** TAG AAT AGC TCG TGG TGC G	34	*trpC^{t}*
IM79	**AGG ACT TCT AGA AGG** AAT AGC GCG GCC TGA CAA	33	pME4564
IM80	**ATT CTT AAT TAA GAT** TGC CGA TCC CAT TCC CCA	33	pME4564
IM81	**ATA TCA GTT AAC GTC** TTT GAA GGT GGT TTG GTT TGG T	37	*^{p}trpC*
IM82	**ACC AGC CCC TGG GTT** GCG CTG CAT GGG CGG TAC	33	*trpC^{t}*
IM83	**AGG ACT TCT AGA AGG** CAG CTG TAG ATC ACT CGA GTT CC	38	pME4564
JST253	GAC GTT AAC TGA TAT TGA AGG AGC AC	26	-
JST254	AAC CCA GGG GCT GGT GA	17	-
ML8	AAA GAA GGA TTA CCT CTA AAC AA	23	-
ML9	TGT ACA GTG ACC GGT GAC	18	-
ML184	**CTC TAG ACC TGG CAT** GGC CAC CGA CTT TAT ATA TCA GT	38	*VDH4*
ML185	**ACC AGC CCC TGG GTT** GTA CCA GCC CGG CTG TG	32	*trpC^{t}*

Primer name	Primer sequence (5' → 3')	Length (-mer)	Overhang to
ML186	**ATA TCA GTT AAC GTC** TTA GAC GCC GAC GGG CTT	33	^{p}*trpC*
ML187	**AGT GGT GAA CTT CAT** TTT GAT GGT TGG GGG TTG GAG A	37	*VDH5*
ML188	**ACC AGC CCC TGG GTT** GCG TCG CGG TTC CCG GA	32	*trpC*^{t}
ML189	**ATA TCA GTT AAC GTC** TTA GGC GGC GAC GCC AAC	33	^{p}*trpC*
RO3	GGT ACC CGG GGA TCT TTC G	19	-
**Primers for quantitative PCR**			
**Primer name**	**Primer sequence (5' → 3')**	**Target gene**	
IM118	GGA ACT TGA GAA GGA GCC TAA G	*EF1α/ Solyc06g005060.3*	
IM119	CAA CAC CAA CAG CAA CAG TCT	*EF1α/ Solyc06g005060.3*	
OLG70 [17]	CAG CGA AAC GCG ATA TGT AG	*5.8S rRNA*	
OLG71 [17]	GGC TTG TAG GGG GTT TAG A	*5.8S rRNA*	
q*VDH1*-F [40]	ACG ATT GCT CTG TTT GCT GGA G	*VDH1/ VDAG_JR2_Chr2g02500a*	
q*VDH1*-R [40]	CCT GGC ACT CTT TGG GGT AGA G	*VDH1/ VDAG_JR2_Chr2g02500a*	
q*VDH2*-F	TGC ACT CCC GTC ATT GCT TAA G	*VDH2/ VDAG_JR2_Chr4g07990a*	
q*VDH2*-R	TCA CCG AAC CGT CTA TCT GAG C	*VDH2/ VDAG_JR2_Chr4g07990a*	
q*VDH3*-F	GAG GAC CGC CAA GTC TAC ATC C	*VDH3/ VDAG_JR2_Chr1g24880a*	
q*VDH3*-R	CAC CCT GGT CAA GAA TGG GAA G	*VDH3/ VDAG_JR2_Chr1g24880a*	
q*VDH4*-F	AAG TCC TTC ACT GCC ATC GTC	*VDH4/ VDAG_JR2_Chr3g09420a*	
q*VDH4*-R	GTT GGC GCT CTT GGG GTT CT	*VDH4/ VDAG_JR2_Chr3g09420a*	
q*VDH5*-F	CCC GTC TGC TGC GCT ACT AA	*VDH5/ VDAG_JR2_Chr1g24730a*	
q*VDH5*-R	GTT GAG GAG GTT GAG GGT GCA G	*VDH5/ VDAG_JR2_Chr1g24730a*	

^{p}: promoter, ^{t}: terminator; bold: overhangs for Seamless Cloning.

Table S4: Plasmids used in this study.

Plasmid name	Description	Reference
pCOM	Cloning vector with *KAN^R*, *^{p}trpC:GEN^R:trpC^t*, left and right border for *A. tumefaciens*-mediated transformation	[41]
pPK2	Cloning vector with *KAN^R* and *HYG^R*; left and right border for *A. tumefaciens*-mediated transformation	[43]
pME4564	Cloning vector with *KAN^R* and *HYG^R*; left and right border for *A. tumefaciens*-mediated transformation	[42]
pME4815	*^{p}gpdA:NAT^R:trpC^t* in pME4564	[42]
pME5484	*^{p}VDH2:^{p}gpdA:GEN^R:trpC^t:VDH2^t* in pME4564	This study
pME5485	*^{p}VDH4:^{p}gpdA:NAT^R:trpC^t:VDH4^t* in pME4564	This study
pME5486	*^{p}VDH5:^{p}gpdA:HYG^R:trpC^t:VDH5^t* in pME4564	This study
pME5487	*^{p}VDH4:VDH4:^{p}trpC:GEN^R:trpC^t:VDH4^t* in pME4564	This study
pME5488	*^{p}VDH5:VDH5:^{p}trpC:GEN^R:trpC^t:VDH5^t* in pME4564	This study
pME5511	*^{p}VDH1:^{p}gpdA:GEN^R:trpC^t:VDH1^t* in pME4564	This study

^{p}: promoter, ^{t}: terminator, *GEN^R*: geneticin resistance marker, *HYG^R*: hygromycin B resistance marker*,* *KAN^R*: kanamycin resistance marker, *NAT^R*: nourseothricin resistance marker.

Table S5: Fungal and bacterial strains used in this study.

Strain name	Description	Reference
Agrobacterium tumefaciens		
AGL1	Strain for *A. tumefaciens*-mediated transformation of *V. dahliae*	[37]
Escherichia coli		
DH5α	Strain for cloning and extraction of plasmids	Invitrogen Thermo Fisher Scientific
Verticillium dahliae		
JR2/WT	*Solanum lycopersicum* isolate	[22]
Δ*VDH2* (VGB648/ VGB649)	*VDH2* deletion strain (Δ*VDH2::*^{p}*trpC:GEN*^{R}*:trpC*^{t})	This study
Δ*VDH4* (VGB566/ VGB567)	*VDH4* deletion strain (Δ*VDH4::*^{p}*gpdA:NAT*^{R}*:trpC*^{t})	This study
Δ*VDH5* (VGB568/ VGB569)	*VDH5* deletion strain (Δ*VDH5::*^{p}*gpdA:HYG*^{R}*:trpC*^{t})	This study
Δ*VDH2/5* (VGB651)	*VDH2/5* double deletion strain (Δ*VDH2::*^{p}*trpC:GEN*^{R}*:trpC*^{t}; Δ*VDH5::*^{p}*gpdA:HYG*^{R}*:trpC*^{t})	This study
Δ*VDH4/5* (VGB623/ VGB624)	*VDH4/5* double deletion strain (Δ*VDH4::*^{p}*gpdA:NAT*^{R}*:trpC*^{t}; Δ*VDH5::*^{p}*gpdA:HYG*^{R}*:trpC*^{t})	This study
Δ*VDH1/4/5* (VGB646/ VGB647)	*VDH1/4/5* triple deletion strain (Δ*VDH1::*^{p}*trpC:GEN*^{R}*:trpC*^{t}; Δ*VDH4::*^{p}*gpdA:NAT*^{R}*:trpC*^{t}; Δ*VDH5::*^{p}*gpdA:HYG*^{R}*:trpC*^{t})	This study
Δ*VDH2/4/5* (VGB657)	*VDH2/4/5* triple deletion strain (Δ*VDH2::*^{p}*trpC:GEN*^{R}*:trpC*^{t}; Δ*VDH4::*^{p}*gpdA:NAT*^{R}*:trpC*^{t}; Δ*VDH5::*^{p}*gpdA:HYG*^{R}*:trpC*^{t})	This study
VDH4-C/ Δ*VDH5* (VGB690/ VGB691)	*VDH4* complementation strain in the *VDH4/5* double deletion background (Δ*VDH4::*^{p}*VDH4:VDH4:*^{p}*trpC:GEN*^{R}*:trpC*^{t}*:VDH4*^{t}; Δ*VDH5::*^{p}*gpdA:HYG*^{R}*:trpC*^{t})	This study
VDH5-C/ Δ*VDH4* (VGB692)	*VDH4* complementation strain in the *VDH4/5* double deletion background (Δ*VDH5::*^{p}*VDH5:VDH5:*^{p}*trpC:GEN*^{R}*:trpC*^{t}*:VDH5*^{t}; Δ*VDH4::*^{p}*gpdA:NAT*^{R}*:trpC*^{t})	This study

^{p}: promoter, ^{t}: terminator, *GEN*^{R}: geneticin resistance marker, *HYG*^{R}: hygromycin B resistance marker, *NAT*^{R}: nourseothricin resistance marker; two VGB numbers for one genotype indicate two independent transformants.

Table S6: Cysteine spacing of *V. dahliae* JR2 hydrophobins and comparison with the consensus of cysteine spacing patterns in class I and II hydrophobins.

**Hydrophobin**	**Cysteine spacing**
Consensus Class I [53]	CX5-7 CCX19-39 CX8-23 CX5 CCX6-18 CX2-13
Consensus Class II [53]	CX9-10 CCX11 CX16 CX8-9 CCX10 CX6-7
Vdh1	CX9 CCX11 CX15 CX6 CCX10 CX6
Vdh2	CX10 CCX11 CX16 CX8 CCX10 CX5
Vdh3	CX8 CCX11 CX16 CX8 CCX10 CX8
Vdh4	CX8 CCX11 CX15 CX8 CCX10 CX6
Vdh5	CX9 CCX11 CX16 CX8 CCX10 CX8
